# Innovations in early detection of chronic non-communicable diseases among adolescents through an easy-to-Use AutoML paradigm

**DOI:** 10.1007/s10729-025-09718-6

**Published:** 2025-08-28

**Authors:** Nevena Rankovic, Dragica Rankovic, Igor Lukic

**Affiliations:** 1 Department of Cognitive Science and Artificial Intelligence, Tilburg University, Warandelaan 2, 5037AB Tilburg, The Netherlands; 2 Department of Informatics, School of Computing, Union University, Knez Mihajlova 5, 11000 Belgrade, Serbia; 3 Department of Preventive Medicine, University of Kragujevac, Svetozara Markovica 69, 34000 Kragujevac, Serbia

**Keywords:** Chronic non-communicable diseases, AutoML paradigm, Tabular variational autoencoder (TVAE) synthetic generation, Model-agnostic/post-hoc methods

## Abstract

In this research, we present an interpretable AutoML approach for the early diagnosis of hypertension and hyperinsulinemia among adolescents, conditions that are critical to identify during these formative years due to their requirement for lifelong care and monitoring. The dataset, collected from 2019 to 2022 by Serbia’s Healthcare Center through an observational cross-sectional study, posed challenges common to medical datasets, including imbalances, data scarcity, and a need for transparent, explainable predictive models. To counter these issues, we utilized three AutoML frameworks - AutoGluon, H2O, and MLJAR - in conjunction with a Tabular Variational Autoencoder (TVAE) to synthetically augment the data points, Prinicipal Component Analysis (PCA) for dimensionality reduction, and SHapley Additive exPlanations (SHAP) and Permutation feature importance analyses to extract insights from the results. AutoGluon outperformed the others on the original dataset, delivering better results with weighted ensemble models for both conditions under a 12-minute budget-time constraint and maintaining all evaluation metrics below a 4% threshold, all without the need for further scaling or calibration in the experimental setup. Our research underscores the broad applicability of the current AutoML paradigm, highlighting its particular benefits for the healthcare domain and diagnostics, where such advanced tools can enhance patient care.

## Highlights

- Three AutoML frameworks were chosen for early diagnose of hyperinsulinemia and hypertension among adolescents.
- Tabular Variational Autoencoder was employed to augment data synthetically.
- Model-agnostic post-hoc methods were used to enhance the explainability of the weighted ensemble models.
- AutoGluon outperformed others on the original dataset with weighted ensemble models under a12-minute budget-time constraint.

## Introduction

The prevalence of stress-inducing factors in modern society is contributing to the emergence and progression of diseases. Hence, alongside lifestyle modifications, regular preventive and diagnostic check-ups become essential. Moreover, healthcare systems must integrate intelligent techniques to enhance their efficacy [1, 2]. The healthcare industry continuously seeks innovative methods to improve healthcare services and outcomes for complex diseases. For instance, hyperinsulinemia, a precursor to type 2 diabetes mellitus, is marked by significantly high insulin levels and can go undetected for years, especially during the pivotal adolescent years. Early detection and management are paramount to prevent subsequent health issues, including metabolic disorders such as hypertension. Adolescence is a critical period for monitoring hyperinsulinemia due to significant hormonal shifts that can affect pancreatic function and insulin regulation. Poor dietary choices and lack of exercise, prevalent among adolescents, exacerbate the risk of metabolic syndromes. Notably, risk factors for hyperinsulinemia include a family history of type 2 diabetes, obesity, insulin resistance, sedentary lifestyle, and poor nutrition [3, 4]. According to the International Diabetes Federation (IDF), hyperinsulinemia is diagnosed when fasting insulin levels exceed 15 $\mu$U/ml, Oral Glucose Tolerance Test (OGTT) results surpass 75 $\mu$U/ml at 120 minutes, or the cumulative insulin level is above 300 $\mu$U/ml. With the rising incidence of hyperinsulinemia during adolescence, the significance of these findings extends to pediatric healthcare. Concurrently, the early diagnosis of hypertension is crucial in a comprehensive approach to combat metabolic syndromes like hyperinsulinemia. Both conditions often coexist and can be asymptomatic in adolescents, yet share common risk factors such as obesity and poor lifestyle habits [5]. Detecting and managing hyperinsulinemia can be an early intervention point for hypertension, averting a series of health issues. Managing hyperinsulinemia through lifestyle changes and medical intervention may also regulate blood pressure, underscoring the interconnection between these conditions. Addressing both hyperinsulinemia and hypertension is crucial for protecting adolescents’ health and preventing the progression of these conditions into adulthood, where they can lead to more severe complications such as cardiovascular disease and type 2 diabetes [6–8].

One area burgeoning with promise is Machine Learning (ML), which has seen enormous expansion in both academic and practical realms globally. However, ML is not universally accessible, transparent, fair, or interpretable for many healthcare institutions. Auto Machine Learning (AutoML) makes ML more accessible by automating many aspects of the ML pipeline, allowing for rapid development and deployment of ML solutions in healthcare [9]. Despite its potential computational inefficiencies, AutoML is poised to enhance sustainability. Greater accessibility can lead to improved sustainability, such as through early disease detection, which minimizes the resources needed for treatment. AutoML presents diverse opportunities for healthcare enhancement, from predicting disease outbreaks and monitoring to identifying high-risk patients and devising personalized treatment strategies. By shifting towards preventive care, AutoML leverages patients’ medical histories and relevant data to recommend treatments, identifying potential health risks before they escalate [10]. Ensemble or hybrid multi-model AutoML frameworks analyze extensive medical datasets to contribute to disease outbreak prediction campaigns or early warning systems. Moreover, AutoML can reveal patterns and correlations that may be missed by human analysis, thus informing more effective disease prevention and management strategies. Additionally, utilizing the most reliable AutoML frameworks and algorithms, healthcare providers can identify high-risk individuals who might be overlooked by traditional risk assessments. This data informs the development of improved care plans, ensuring that high-risk patients receive the attention necessary to manage their conditions effectively. Furthermore, SHAP (SHapley Additive exPlanations) and Permutation Feature Importance are analytical methods that will be leveraged to enhance the interpretability and transparency of AutoML frameworks applied to hyperinsulinemia and hypertension diagnostics [11, 12]. SHAP values elucidate the contribution of each feature to the predictive model by drawing on cooperative game theory, while Permutation Feature Importance assesses the impact of feature shuffling on model accuracy, providing insights into feature relevance [13]. Together, they will offer a comprehensive understanding of how the AutoML models make diagnostic predictions, which is essential for clinical validation and trust in automated medical decision-making [14, 15]. Ultimately, while the integration of AutoML into healthcare systems does involve using complex *’black-box’* models, our focus is on achieving fast and accurate results that are practical and easy to use for healthcare professionals. We recognize the interpretability concerns, but we aim to bridge this gap by providing transparent, user-friendly tools that deliver the benefits of advanced machine learning without requiring deep technical understanding. This approach allows healthcare providers to leverage the power of these models effectively, ensuring both accuracy and ease of use in clinical settings. This advancement promises significant improvements in healthcare quality and treatment outcomes for complex diseases, while also revealing healthcare gaps and risk categories that have previously gone unnoticed.

Therefore, the primary Research Objectives (ROs) guiding our study, along with contributions behind them are as follows:

1. Evaluate the extent to which AutoML can improve classification accuracy for hyperinsulinemia and hypertension diagnostics when a feature dimensionality reduction technique is applied.
2. Determine the efficacy of employing a Tabular Variational Autoencoder (TVAE) for synthetic data generation in enhancing the classification performance of hyperinsulinemia and hypertension.
3. Define interpretability metrics and use SHAP and Permutation Feature Importance as post-hoc methods to gain insights into the most informative and contributing features of the best-performing diagnostic model.

Follwing our ROs, the primary motivation for our study is the development of an interpretable AutoML approach for the early diagnosis of hypertension and hyperinsulinemia in adolescents. These conditions are critical to identify during formative years due to the necessity for lifelong care and monitoring. Our research relies on data collected from 2019 to 2022 at the Health Center of Serbia through an observational cross-sectional study, which faces common medical data challenges, including imbalances, data scarcity, and the need for transparent, explainable predictive models. The goal is to overcome these issues by using AutoML frameworks such as AutoGluon, H2O, and MLJAR, in combination with a TVAE for synthetic data augmentation, PCA for dimensionality reduction, and SHAP and Permutation Feature Importance to extract insights from the results. Our research highlights the broad application of the current AutoML paradigm, emphasizing its specific benefits for healthcare and diagnostics, where such advanced tools can significantly improve patient care.

The structure of the paper is organized as follows: Section 2 addresses the most recent research in the field medical diagnostics using machine learning or deep learning models with various experimental settings. Section 3 is devoted to the methodology setup of this research. Section 4 presents the research findings. Section 5 discusses the obtained results, including potential limitations and future directions in the field. Concluding remarks are provided in Section 6.

## Related work

In this section, we will explore the latest research in the medical domain, focusing on Machine Learning and Deep Learning models, both individually and in ensemble or hybrid forms, used to examine different chronic non-communicable diseases. We will also review the characteristics of the settings employed for the experimental setup of the chosen models. Additionally, we will investigate whether recent studies have employed any type of post-hoc/model-agnostic or model-specific methods to provide the most informative insights into the internal mechanisms of their chosen settings.

### Machine learning for medical diagnostics

Global health costs are largely attributable to chronic non-communicable diseases (CNCDs). Individuals with these conditions require lifelong care. Since each strategy has benefits as well as drawbacks, there are no set standards for identifying the most effective one in real-time clinical practice [16, 17]. Support Vector Machines (SVM), Logistic Regression (LR), and clustering were the most often employed techniques among those examined. These models are predicted to have a greater impact in medical practice in a short time because they are particularly helpful for diagnosing and classifying CNCDs [16]. In a comparative study involving 6,762 Asian adults, five ML models were evaluated against standard logistic regression for predicting chronic disease risk. Despite the variety of models tested-including neural networks, support vector machines, random forests, gradient boosting machines, and k-nearest neighbors-the performance gap was marginal and statistically nonsignificant. The study concluded that logistic regression performs comparably to complex ML models, with the difference of only 1%, especially for diseases with low incidence and straightforward clinical predictors [18]. The authors in [19] applied eight varied machine learning models—including Logistic Regression, K-Nearest Neighbours, Support Vector Machine, Naïve Bayes, Decision Tree, Random Forest, XGBoost, and Artificial Neural Network - to forecast the risk of chronic diseases from selected features. In-depth experiments from this and another study by [20] showed classifier performance, with the Area Under Curve (AUC) ranging from 0.79 to 0.91, and highlighted the Random Forest model as the most effective among them. Significantly, features such as eigenvector centrality, closeness centrality of the network, and patient age were determined to be pivotal, underscoring the considerable potential of their model in enhancing healthcare services. The research conducted in [21] presents the use of machine learning as an emerging approach for computational diabetes diagnosis, indicating a necessity for increased precision in predictive models. It proposes a machine learning framework that utilizes the PIMA Indian dataset [22] and the LMCH diabetes dataset, aimed at enhancing classification performance through feature selection and missing value imputation. To optimize features, the framework incorporates Spearman correlation and polynomial regression, and introduces a novel twice-growth deep neural network (2GDNN) model, with model hyperparameters refined via grid search and stratified k-fold cross-validation. This approach yields a robust machine learning model that surpasses existing benchmarks in the prediction and diagnosis of diabetes mellitus. Between January 2018 and May 2021, 21 articles were evaluated, focusing on aspects such as variable selection, train-test split, data balancing, outcome definition, chosen algorithms, and performance metrics. The studies reported an Area Under the Receiver Operating Characteristic curve (AUROC) ranging from 0.766 to 1.00. Support Vector Machines, Extreme Gradient Boosting (XGBoost), and random forest were the algorithms most commonly recognized for their superior performance [23]. In [24] researchers utilized the Cleveland heart disease dataset from the UCI repository, which contains 303 instances and 76 attributes, of which 14 were deemed significant for evaluating the performance of various algorithms. The study aimed to predict the likelihood of heart disease in patients. Notably, the K-nearest neighbor algorithm was identified as yielding the highest accuracy score in their findings. Therefore, investigating and monitoring blood pressure levels is highly important [25]. Finally, in a comprehensive review by [26] it has been found that ML algorithms have been applied in high blood pressure studies following two approaches: hypertension stage classification based on clinical data and blood pressure estimation based on related physiological signals, along with different feature selection techniques.

### Deep learning for medical diagnostics

Chronic non-communicable diseases pose a major risk to human health, making it crucial to forecast their onset before actual diagnosis, allowing for timely and effective treatment [27]. This study introduces a novel approach to predicting multiple chronic diseases simultaneously, transforming the issue into a multi-label classification challenge. A new convolutional neural network model, GroupNet, is designed specifically for this task, utilizing Binary Relevance and Label Powerset to handle the multi-faceted nature of chronic conditions. GroupNet incorporates a unique correlated loss function that takes into account the interrelations between different diseases to improve prediction accuracy. Tested against other models on data from a local medical center, GroupNet achieved superior performance, reaching an accuracy of 81.13% [27]. Additionally, one study evaluated various deep learning models like CNNs, LSTMs, and hierarchical structures, demonstrating their superior performance over traditional models by effectively interpreting raw text, numerical values, and negations without requiring disease-specific features, with analysis on a patient cohort of approximately one million. Furthermore, the authors explored diverse visualization techniques to aid medical professionals in understanding model predictions [28]. The research conducted in [29] presents advancements in the prediction of chronic diseases through deep learning. The study showcases an enhanced model leveraging deep learning techniques with specific activation functions like ReLU and sigmoid, and an Adam optimizer. This approach is compared against traditional machine learning methods like K-nearest neighbor and decision trees. The results indicate a significant improvement in prediction accuracy using deep learning, achieving up to 98.3% accuracy, surpassing conventional algorithms. The goal is to accurately forecast chronic diseases at an individual level by utilizing the power of deep learning within the healthcare domain. Utilizing neural network models, the authors predicted chronic diseases and risk factors such as hypertension, hyperglycemia, and dyslipidemia from 1222 retinal fundus images and associated anthropometric and biochemical data from a study in Xinxiang County, China. The models showcased high predictive accuracy, with AUCs of 0.880 for hyperglycemia, 0.766 for hypertension, and 0.703 for dyslipidemia, and were also effective in forecasting various erythrocyte parameters and cardiovascular risk factors with AUCs over 0.7. This indicates the viability of deep learning for the prediction of a range of chronic conditions and their risk factors [30]. In [31] the authors employed three deep neural network models to assess the risk of type 2 diabetes mellitus, addressing the common challenge of imbalanced data in medical AI which can skew results if overlooked. To avoid the pitfalls of relying solely on accuracy, they evaluated the models using precision, recall, AUC, and AUPRC, and investigated the overall significance of different variables in T2DM risk. Incorporating personality features and utilizing Shapley Values, they personalized the model’s predictions for individual patients’ prognoses. In the research conducted by [32], an leveraged a medical deep-learning model paired with a newly developed highly sensitive biosensor to advance the detection of diabetes, greatly refining diagnostic accuracy and efficiency. This innovation is poised to transform diabetes management by reducing costs and enhancing accessibility, promising a significant impact on patient care. The proposed model has demonstrated exceptional performance metrics including 96.21% accuracy, 91.53% precision, and an impressive diagnostic odds ratio of 89.90%. The comprehensive review of current studies presented in [33, 34] highlights that deep learning methods have surpassed traditional machine learning in numerous diabetes-related applications, setting new benchmarks for performance. However, challenges such as limited data access and the need for clearer model interpretation remain prevalent, as they are vital for the medical field.

### Post-hoc methods for interpretable medical diagnostics

To better understand and derive the most insightful feedback from the selected models, it’s crucial to grasp both the results they produce and their internal workings. For every classification domain, identifying which features significantly influence the final decision, as well as the most impactful concepts is essential [33]. Conversely, it is equally important to recognize features that may remain unchanged and not affect the final decision-making process for the chosen models. Clarifying which features are pivotal will enhance our interpretation of the model’s functionality [19]. To understand the nuanced contributions of each feature to predictive models, calculating Shapley values is vital as they provide a fair distribution of contribution across features. To refine the model and train the neural network accordingly, and to articulate the significance of feature importance as it varies by individual disease risk factors. For example, in [31] to consider BMI a more decisive factor than age for younger individuals at risk of T2DM, while for older individuals, to regard age as more significant than BMI, highlighting the concept of individual feature importance. To employ Shapley values in personalizing the model for each new instance, and to demonstrate through a hypothetical case how each feature shifts the disease risk based on its importance to that person, thus exploring the risk of T2DM tailored to individual characteristics. In the research conducted by [35], the application of a Random Forest algorithm to the dataset yielded predictions with 76% accuracy, edging out the 74% accuracy rates of both the support vector machine and XGBoost. Despite glucose being the most significant predictor, the modest accuracy indicates that these models may not yet be robust enough for real-world applications.Additionally, the study determined the importance of each dataset feature by averaging their marginal contributions across all possible feature combinations used in the model. Through this method, the impact of adding or removing a feature on model accuracy was meticulously assessed. The dataset was expanded from 8 to 36 variables, whereupon a tree-based machine learning algorithm, specifically XGBoost, was utilized due to its speed and relative precision. The model was fine-tuned to consider attributes with a Shapley value of 0.1 as critical for prediction, focusing on the top 15 predictors identified by SHAP through the XgBoost framework. Moreover, they concluded that glucose by far contributed more than other attributes with 0.990 values followed by BMI 0.702. The attribute BMI is followed closely by Age, and clearly shows that the attribute diabetes degree function contributes more than the age of the patient in the prediction, where in other study [36] SHAP indicated that Hemoglobin (Hb), age, total bile acids (TBA) and lipoprotein(a)(LP-a) are the top four important risk factors for peripheral vascular disease in type 2 diabetes mellitus. Also, without SHAP, the attribute pregnancies have less impact than age but from our plot and analysis, it is almost twice as impactful as age to the prediction. The increase in the accuracies of the models to this appropriate attachment of importance and weight to each of the attributes. Ultimately, interpretability tools like SHAP are essential for assessing predictive models in the context of chronic, non-communicable disease diagnostics. They are especially critical when these models inform decision-making or control functions where patient safety is paramount. Consequently, integrating these tools into standard model evaluation processes is highly advisable for enhanced reliability and safety [37, 38].

As it becomes evident from the available literature, Machine Learning and Deep Learning algorithms are powerful resources for enhancing preventive clinical decisions and specific public health policies for hyperinsulinemia and hypertension. Nevertheless, based on our present knowledge and the research available, several shortcomings have been identified that must be addressed:

- Technical aspects such as outcome definition;
- Availability of the final code;
- Predictive performance;
- Explainability for obtaining more informative feedback;
- Data leakage, which needs consistent and critical evaluation;
- A focus in most studies on diabetes rather than on its early latent stage– hyperinsulinemia;
- Absence of testing for all functionalities that AutoML can offer, for example, 662 ensemble models were tested;
- AutoML’s methodology of training, tuning, and stacking a variety of models, as opposed to traditional data science methods that select specific hyperparameters to fine-tune.

## Methodology

In this section, we will describe three AutoML frameworks to be used for detecting hyperinsulinemia or hypertension condition from a medical dataset collected by a Healthcare center in Serbia, using data for adolescents aged 12 to 17 years. AutoML is a promising method for this dataset due to its demonstrated performance in detecting other diseases and conditions [39–41] as well as the dataset’s small size, which reduces computational difficulty [9]. First, we will delve into the characteristics of the adolescents’ dataset. Second, the method and reasoning for generating synthetic data will be discussed. Third, the AutoML frameworks to be implemented in the research will be elaborated upon. Then, we shall discuss the experimental design of the research. Fifth, the evaluation metrics will be briefly described. Finally, the environment in which the experiment will run will be detailed.

### Exploratory data analysis (EDA)

**Fig. 1:**
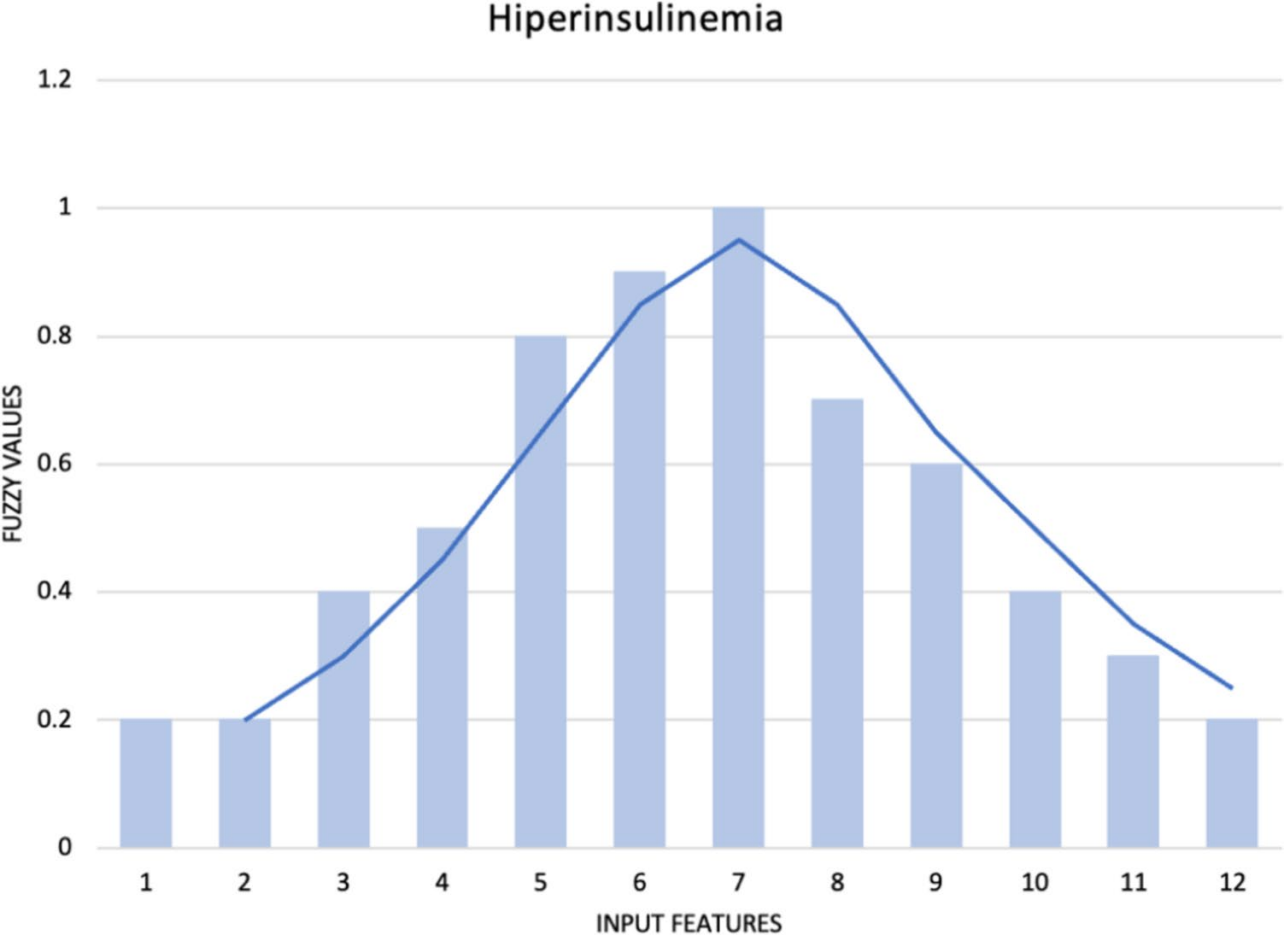
Distribution of the target variable: hyperinsulinemia

**Fig. 2:**
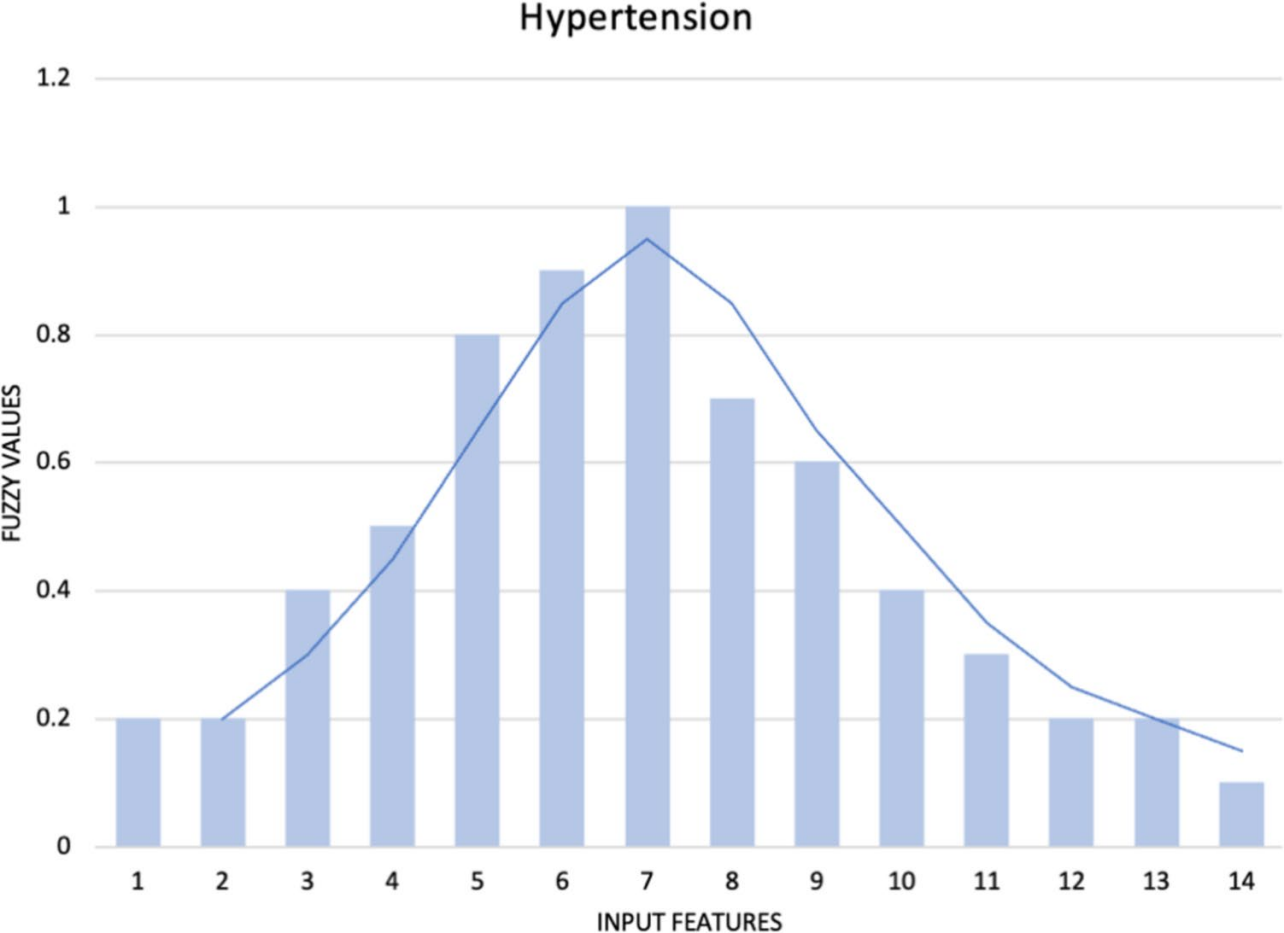
Distribution of the target variable: hypertension

The dataset was collected on a national level by the Healthcare Center in Serbia during mandatory school health examinations. The dataset comprises seven groups of features: the first group pertains to the basic characteristics of the participants (gender, age, height, weight, waist circumference, systolic blood pressure, diastolic blood pressure, and pulse); the second group encompasses the results of hematological and biochemical analyses (white blood cells, red blood cells, hemoglobin, hematocrit, MCV, MCH, MCHC, RDW, platelets, segmented, MID, lymphocytes, sedimentation rate, CRP, glucose, HDL cholesterol, LDL cholesterol, triglycerides, urea, creatinine, total proteins, total bilirubin, AST, ALT, sodium, potassium, chloride); the third group includes the assessment of dietary habits using the YAQ questionnaire with 20 different questions about the quantity and type of consumption of various foods and beverages; the fourth group involves the assessment of physical activity using the IPAQ questionnaire with 10 different questions; the fifth group assesses the history of hereditary diseases in close and extended family using the FHQ questionnaire with 10 different questions; the sixth group assesses the consumption of alcohol, cigarettes, and other psychoactive substances using the BRFSS questionnaire; and the seventh group relates to socio-demographic conditions and characteristics such as living conditions, learning and engaging in free-time activities, parents’ education, parents’ employment, and family structure. A total of 93 features were considered. For the assessment of dietary habits, physical activity, and the consumption of alcohol, cigarettes, and other psychoactive substances, a Likert scale was employed. To mitigate bias within the dataset, individuals—both healthy and those diagnosed with hyperinsulinemia or hypertension—were selected from comparable backgrounds. Efforts were made to ensure an equitable distribution concerning age and gender across all adolescent participants. The dataset has 336 instances and is a well-balanced dataset [42] Figs. 1 and 2, with 160 instances of hyperinsulinemia, 176 healthy instances, 145 instances of hypertension, and 196 health instances. The dataset has less than 5% of missing values, so they were removed. Additionally, the Figs. 1 and 2 provided represent the distribution of the target variables for hyperinsulinemia and hypertension, respectively. These well-balanced datasets illustrate that the data points are evenly distributed across the range of input features, ensuring that the models can learn effectively without being biased towards any particular segment of the data. Given the varying nature of the features analyzed in these datasets, normalization was applied to scale the input features. This step is crucial as it brings all features onto a comparable scale, which is particularly important when using machine learning algorithms that are sensitive to the scale of input data. Moreover, the smooth curves overlaid on the histograms provide a clear visualization of the distribution shape, further confirming that the datasets are well-prepared for subsequent analysis. This balanced and normalized data ensures that the AutoML frameworks, such as AutoGluon, H2O, and MLJAR, can efficiently process and generate accurate predictions for these non-communicable diseases.

These specific data points were also intentionally not substituted with the value of 0. This decision aligns with the guidance of medical experts who assert that, in the context of this attribute type, 0 is not a valid or appropriate value. Considering that, for instance, laboratory analysis parameters are provided in diverse parameter units for all input features, it becomes imperative to employ the min-max normalization scaling technique within a specific narrow range [0, 1] [43]. This ensures the creation of a new scaled dataset based on the original, effectively harmonizing the varied parameter units. Principal Component Analysis (PCA) was utilized as a dimensionality reduction technique to refine the initial set of 93 features to the most pertinent ones for input into the selected AutoML frameworks. This method not only streamlines the dataset for more efficient processing but also aids in reducing the likelihood of overfitting by removing superfluous variables. Moreover, transforming the data into a set of linearly uncorrelated principal components, with PCA we are concentrated on examination of the data’s inherent structure, thus improving the predictive capacity of the models derived from this condensed dataset. The PCA analysis transforms the original values of variables into principal components together using the following formulas Eqs. 1, 2 [44]:

1 $$\begin{aligned} Y = X \cdot C \end{aligned}$$

*X* is a set of *n* vectors, ($x_{1}$,...,$x_{n}$) where each $X_i$ element represents an instance of our dataset, and *C* is a square matrix of order *n*:

2 $$\begin{aligned} C_{ij} = \begin{bmatrix} c_{11} & \cdots & c_{1n} \\ \vdots & \ddots & \vdots \\ c_{n1} & \cdots & c_{nn} \end{bmatrix}, \quad Y_{ij} = \begin{bmatrix} x_{1} \\ \vdots \\ x_{n} \end{bmatrix} \cdot C = \begin{bmatrix} y_{1} \\ \vdots \\ y_{n} \end{bmatrix}, \end{aligned}$$

where $$i=\overline{1,n}$$, $$j=\overline{1,n}$$.

When interpreting principal components, it is often useful to know the correlations of the original variables with the principal components. The correlation between variable $X_i$ and principal component $Y_j$ is, formulas Eqs. 3, 4:

3 $$\begin{aligned} K_{ij} = \sqrt{\frac{c_{ij}^2 \cdot \textrm{Var}(y_j)}{\sigma _{ii}^2}} \end{aligned}$$

where $\sigma _{ii}^2 = \frac{1}{n-1} \sum _{i=1}^{n} (x_i - \bar{x})^2$, and the mean value $\bar{x}$ is calculated as:

4 $$\begin{aligned} \bar{x} = \frac{\sum _{i=1}^{n} x_i}{n} \end{aligned}$$

PCA represents eigenvectors and eigenvalues of the covariance matrix. Eigenvectors will determine the directions of the new feature space, while eigenvalues will determine the magnitudes.

From $(\lambda I - C)X = 0$, in other words: $CX = \lambda X$, we define a set $S$ of all vectors that satisfy this equation as, formula Eq. 5:

5 $$\begin{aligned} S = \{x| (\lambda I - C)X = 0\}. \end{aligned}$$

The small size of the dataset could pose a challenge when training various models, particularly deep learning models, which require a substantial amount of data to perform well and to avoid overfitting [45]. To address this, we will outline the measures undertaken to augment the available data synthetically, while still preserving the statistical properties of the original dataset. As already mentioned, the dataset consists of 336 instances with 93 features, including 160 instances of hyperinsulinemia and 176 healthy instances, as well as 145 instances with hypertension and 191 healthy instances. Exploratory data analysis (EDA) for the features identified as relevant after performing the PCA can be found in Tables 1 and 2 for each of the observed target variables hyperinsulinemia an hypertension, respectively. To note, in this study, predictor variables were collected at a single, standardized medical assessment per patient, ensuring a uniform data collection framework. The corresponding hyperinsulinemia labels were assigned based on diagnostic evaluations conducted during the same visit or the immediate follow-up assessment within a fixed clinical cycle. This methodology ensures consistency in label assignment and minimizes temporal discrepancies that could otherwise introduce bias. While our model does not incorporate real-time dynamic monitoring, it effectively captures longitudinal risk through structured observational data, making it applicable for both risk stratification and predictive analytics in clinical settings.

**Table 1:** EDA of input features of hyperinsulinemia target

No.	Risk Factor	Mean±SD	Min	Max
1	Glucose (mmol/l)	8.4±2.6	5.8	11
2	BMI (kg/m$^{2}$)	27.1±4.3	22.8	31.4
3	LDL Cholesterol (mmol/l)	3.92±1.7	2.2	5.6
4	Insufficient physical activity	2.2±1.6	1	5
5	Improper diet	2.7±1.4	1	5
6	Genetic predispositions for type 2 diabetes	3.1±1.3	1	5
7	Systolic blood pressure (mmHg)	148.6±16.4	134.2	163
8	Diastolic blood pressure (mmHg)	98.1±11.5	86.6	99.6
9	Stress	3.2±1.1	1	5
10	Alcohol consumption	3.5±1.6	1	5
11	Cigarette consumption	3.2±1.3	1	5
12	Consumption of psychoactive substances	2.7±0.8	1	5

$^{*}$where SD=Standard Deviation

**Table 2:** EDA of input features of hypertension target

No.	Risk Factor	Mean±SD	Min	Max
1	Systolic blood pressure (mmHg)	148.6±16.4	134.2	163
2	Diastolic blood pressure (mmHg)	98.1±11.5	86.6	99.6
3	BMI (kg/m$^2$)	27.1±4.3	22.8	31.4
4	LDL Cholesterol (mmol/l)	3.92±1.7	2.2	5.6
5	Insufficient physical activity	2.2±1.6	1	5
6	Improper diet	2.7±1.4	1	5
7	Genetic predispositions for cardiovascular disease	3.3±1.4	1	5
8	Glucose (mmol/l)	8.4±2.6	5.8	11
9	Stress	3.2±1.1	1	5
10	Alcohol consumption	3.5±1.6	1	5
11	Cigarette consumption	3.2±1.3	1	5
12	Consumption of psychoactive substances	2.7±0.8	1	5
13	Sleep disorder	2.4±1.4	1	5
14	Urinary problems	2.2±1.1	1	5

$^{*}$where SD=Standard Deviation

### Synthetic data generation

In this section we will describe the steps that were taken in order to generate synthetic data and artificially increase the original dataset tailoring the statistical characteristics. The relatively small sample size in our dataset may lead to compromised model performance. Limited data can yield subpar results in certain models, notably those based on deep learning. Additionally, there’s an increased likelihood of models overfitting to the training data. Synthetic data generation offers a viable remedy to this issue [46–48]. In healthcare, where amassing ample data for machine and deep learning models is challenging, synthetic data creation has gained traction. Moreover, synthetic data serves as a method to anonymize sensitive information [49]. Synthetic data generation enables the artificial expansion of training datasets. The core concept is to produce data that mirrors the real dataset’s properties, aiming for indistinguishability [47]. To create synthetic data, one must develop a model capable of identifying and replicating data patterns to generate new, synthetic instances. Techniques like Variational Auto Encoders (VAE) and Generative Adversarial Networks (GAN) have been effective for synthesizing data from tabular datasets [47]. Research on synthetic data generation has primarily focused on datasets larger than the our dataset featured in this paper. Nonetheless, evidence suggests these techniques are effective even with smaller datasets. Employing Deep Learning methods like VAEs and GANs for synthetic data production has been associated with enhanced model performance and reduced overfitting [47, 48]. Research on synthetic data generation within healthcare [46] has identified various models suited for this task. Among them, Conditional Tabular Generative Adversarial Network (CTGAN), Tabular VAE (TVAE), and TableGAN stand out [48, 50]. In scenarios with smaller datasets, TVAE outperformed others in producing synthetic data beneficial for training Machine Learning and Deep Learning models. This was assessed by the synthetic data’s statistical resemblance to the original and its efficacy in model training [48]. Consequently, this paper will employ the TVAE model to generate synthetic data based on its effectiveness with limited datasets.

#### Tabular variational autoencoder (TVAE)

The Tabular Variational Auto Encoder (TVAE) model modifies the broader concept of autoencoders, which are unsupervised neural networks designed to reproduce their input as their output. Comprising an encoder and a decoder, the encoder transforms the input into a new state called the latent vector, which the decoder then converts back to a form resembling the input. The model is trained to mirror the input-output relationship [51]. VAEs serve as a variant of autoencoders, differing primarily in that they aim to learn the probability distribution characterizing the training data. By generating a vector of means and standard deviations, VAEs establish a continuous latent space. This space allows for the generation of new samples that mirror the training data, making VAEs adept at creating synthetic data from numerical information [51]. Moreover, the TVAE model, specifically engineered for the creation of synthetic tabular data, demonstrates high efficiency with smaller datasets by producing data closely resembling the original. The TVAE generated synthetic data related to hyperinsulinemia and hypertension diagnostics, producing 10000 synthetic samples within 500 epochs for each dataset. This makes it particularly well-suited for training both machine learning and deep learning models, setting it apart from other synthetic data generators [48]. While sharing similarities with the conventional VAE, the TVAE distinguishes itself by its capability to process and replicate categorical data found in real tabular datasets. For continuous variables, it employs Gaussian distributions, and for categorical variables, it utilizes probability mass functions [52].

#### Synthetic data evalution

To be utilized alongside training data for AutoML models, synthetic data must closely mirror real training data. The Kolmogorov-Smirnov test is proposed to assess the similarity between the real and synthetic data by analyzing the columns, a method extensively cited in research for comparing real and synthetic datasets [47, 48, 50, 51]. This test evaluates the closeness of the cumulative distribution functions (CDFs) of real and synthetic data, with the Kolmogorov-Smirnov statistic representing the maximum divergence between these CDFs, on a scale from 0 to 1. An inverted Kolmogorov-Smirnov statistic is utilized in this study, where a score of 1 signifies identical data columns, and 0 represents completely divergent columns [48]. Additionally, this research will measure the correlation between real and synthetic data columns to determine if the synthetic data replicates the inter-variable correlations found in the real data, using Pearson’s correlation coefficient for each column pair. The TVAE model and evaluation metrics will be deployed using the Synthetic Data Vault package [53]. The results of the K-S test showed that the inverted Kolmogorov-Smirnov statistic scores were consistently close to 1, approximately greater than 0.9, indicating a high degree of similarity between the synthesized data and the original data columns. This high similarity suggests that the TVAE model effectively captured the distributions of the original data, supporting the validity of the synthetic data generated. These results are crucial as they validate that the synthetic data closely mirrors the real training data, making it suitable for use in training AutoML models. Moreover, the strong correlation between real and synthetic data columns, confirmed by Pearson’s correlation coefficients, indicates that the inter-variable relationships were preserved, further enhancing the reliability of the synthetic data for diagnostic purposes.

### AutoML frameworks

**Fig. 3:**
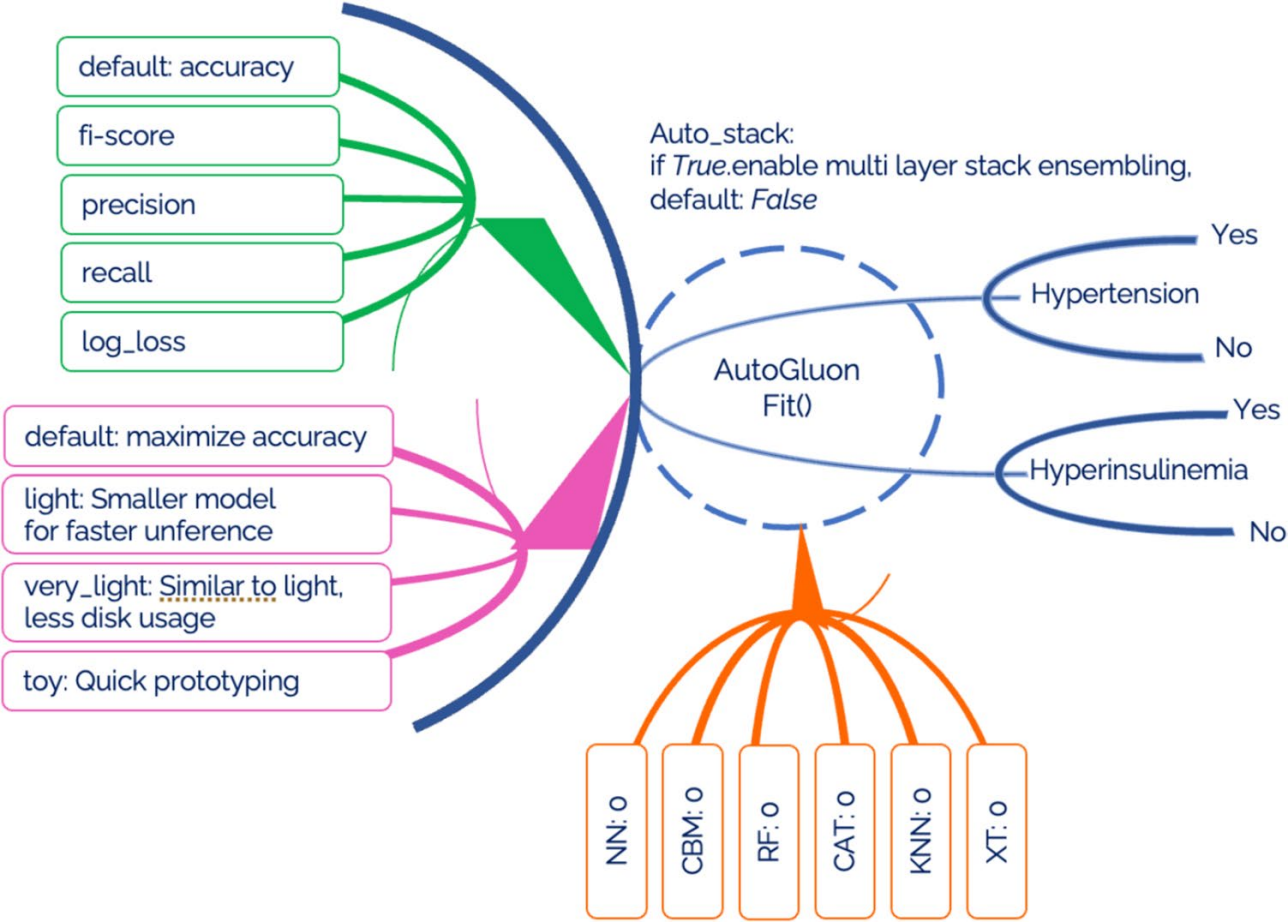
AutoGluon pipeline

In this research we will use three AutoML frameworks: AutoGluon-Tabular, H20 and MLJAR. The first chosen AutoGluon, an open-source framework for automated machine learning (AutoML), facilitates the rapid and straightforward construction of machine learning pipelines by its users. It demonstrates competence in handling tabular data, executing numerous stages of the machine learning workflow with minimal code [54]. Moreover, AutoGluon distinguishes itself as a framework capable of achieving consistently high performance across a diverse array of tasks [55]. Comparative analyses with other AutoML frameworks reveal that AutoGluon emerges as the superior model in overall performance. AutoML frameworks adopt various methodologies to address the Combined Algorithm Selection and Hyperparameter optimization (CASH) problem. Unlike pursuing innovative strategies for solving the CASH dilemma, AutoGluon concentrates on employing a predetermined suite of models with preset hyperparameters, established through collaboration with expert data scientists. This approach is predicated on the observation that the exhaustive exploration of model and hyperparameter permutations during the CASH problem-solving process often includes configurations that seasoned practitioners would typically overlook. By adopting these predetermined hyperparameters, AutoGluon enhances its computational efficiency relative to other AutoML frameworks, without compromising on its competitive edge in performance [54, 55]. Furthermore, AutoGluon implements an automatic, model-agnostic preprocessing of data, adeptly identifying the nature of data columns as numerical, categorical, text, or date/time. Data classified as uncategorized, which pertains to non-numeric and non-informative content, is subsequently excluded from the dataset. Depending on the data type, specific transformations are applied to text and date-time information. However, it is noted that in the context of the cited research, only numerical data was utilized, thus no transformations were necessitated during the model-agnostic preprocessing phase [54]. Following this, AutoGluon progresses to model-specific preprocessing, where the feature set derived from the model-agnostic phase is tailored according to the requirements of individual models. For instance, in the case of neural networks, this stage involves the imputation of missing values using the median, application of quantile normalization to address skewed distributions, and the normalization of remaining numerical variables to achieve a mean of zero and unit variance. During the training process, AutoGluon employs a strategic sequencing of model deployment, prioritizing those models historically recognized for their robust performance before considering more context-specific models. This sequencing approach is particularly advantageous in scenarios with limited time, ensuring that models with a higher likelihood of success are addressed initially. The repertoire of models under AutoGluon’s consideration includes neural networks, LightGBM boosted trees, CatBoost boosted trees, Random Forests, Extremely Randomized Forests, and k-Nearest Neighbours. Specifically, the neural network architecture utilized by AutoGluon is a feed-forward configuration, incorporating dense layers, batch normalization, and dropout mechanisms. It is noteworthy that the neural network’s architecture is not subject to optimization during the training phase. Instead, adjustments to the architecture are made in response to the characteristics of the training data, allowing for a dynamic restructuring based on the dataset’s dimensions [54]. Within the architecture of AutoGluon, support for multi-layer stacking is incorporated, serving as a mechanism to augment performance through the integration of outputs from various models into a new, composite model. Multi-layer stacking further extends this concept by building upon the initial stacked models, effectively utilizing them as foundational layers for the creation of complex, multi-tiered models. The construction of such multilayered stacking models is inherently resource-intensive. To mitigate computational demands, AutoGluon optimizes this process by reutilizing the foundational models along with their predetermined hyperparameters. A distinctive feature of AutoGluon’s implementation is the transmission not only of model predictions but also of the corresponding data. This enables subsequent models to leverage this data, refining their predictions with greater precision [54]. Moreover, AutoGluon incorporates the technique of repeated k-fold bagging across all model tiers. This method, employed to diminish the variance in the predictions of ensemble models, involves dividing the dataset into k subsets via stratified random sampling. Subsequently, k models are trained, each excluding a different subset for validation, thus ensuring out-of-fold predictions for each data point in the training set. By repeating this process multiple times, AutoGluon enhances the robustness of the method against overfitting, particularly beneficial for smaller datasets. AutoGluon initiates the training process by allocating a predefined time budget to each model, a strategy that ensures adherence to user-defined temporal limitations. Should the anticipated training duration of a model surpass its allocated budget, the model’s training is forfeited. This approach allows AutoGluon to operate effectively within set time constraints, consistently delivering commendable performance without necessitating hyperparameter optimization, largely attributed to the strategic model stacking approach [54]. An overview of the AutoGluon pipeline can be seen in Fig. 3.

**Fig. 4:**
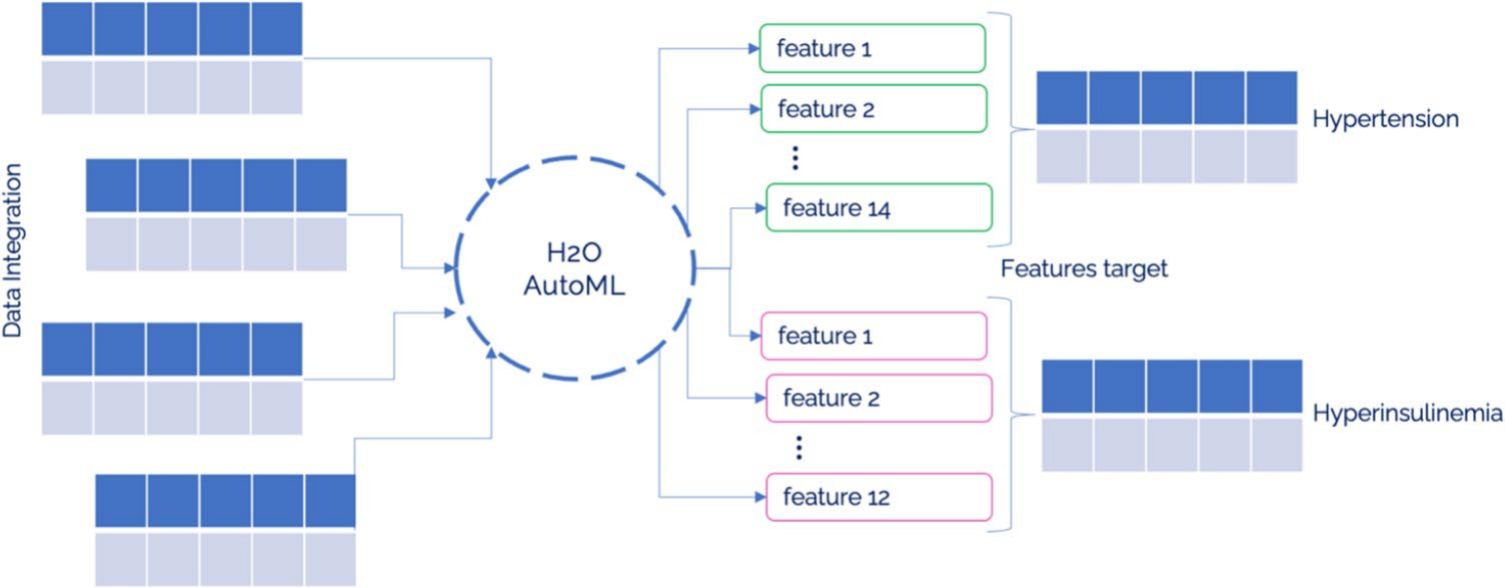
H20 pipeline

The second chosen H2O AutoML, embedded within the broader H2O framework, stands as a prominently utilized and efficacious Automated Machine Learning (AutoML) system, noted for its widespread adoption and high performance [55, 56]. Recognized as one of the foremost AutoML platforms, H2O AutoML is characterized by its open-source nature and scalability, facilitating rapid predictions post-training [54]. In its approach to the Combined Algorithm Selection and Hyperparameter optimization (CASH) problem, H2O AutoML, akin to AutoGluon, refrains from implementing intricate algorithms. Instead, it selects a finite array of models within a defined hyperparameter space for optimization. The selection of these models and their corresponding search spaces is informed by benchmark experiments across various datasets and consultation with expert data scientists. Through the employment of random search techniques within these established parameters, H2O AutoML ascertains the most favorable hyperparameter configurations, enabling it to remain competitive against other AutoML frameworks that may utilize more elaborate algorithms [56]. Current iterations of H2O AutoML provide limited automatic data preprocessing capabilities, maintaining the preprocessing functionalities available in the standard H2O framework. These functionalities encompass automatic data imputation, normalization as necessary, and one-hot encoding specifically for XGBoost models. Users are required to undertake preliminary data preprocessing steps; initially, data must be imported as an H2O dataframe. Subsequently, it is imperative to ensure all variables conform to the framework’s acceptable formats. Given that the Darwin dataset exclusively comprises numerical features, no further preprocessing was requisite in this instance. H2O AutoML integrates a comprehensive range of models within its training regimen, encompassing XGBoost Gradient Boosting Machines, H2O Gradient Boosting Machines, both Random Forest and Extremely Random Forest, Deep Neural Networks, and Generalized Linear Models. The training methodology unfolds in two primary stages: initially, all models undergo training employing default hyperparameter configurations, followed by a subsequent phase where models are refined using random search within a specified hyperparameter search space. The sequence of model training is deliberately structured, commencing with models that have historically demonstrated superior performance, before broadening the focus to include a more varied array of models. This strategy aims to enhance the overall performance of the resulting ensemble models. Subsequent to the initial training phases, the process advances to the construction of stacked ensemble models, a move aimed at amplifying the predictive accuracy of the composite model. Two distinct types of stacked ensemble models are developed: one comprising solely the highest-performing model from each category, and another that amalgamates all trained models. These ensemble models are then refined through a meta-learning algorithm known as the super learner, which allocates and optimizes weights for the models within the ensemble, utilizing a dedicated training set for this purpose. It is crucial to note that during this optimization phase, the individual models remain unaltered; the focus is exclusively on the adjustment and training of the associated weights [56, 57]. This approach to ensemble model creation not only mitigates the risk of overfitting but also diminishes variability, offering a marked improvement over the performance of individual models. An overview of the H20 pipeline can be seen in Fig. 4.

**Fig. 5:**
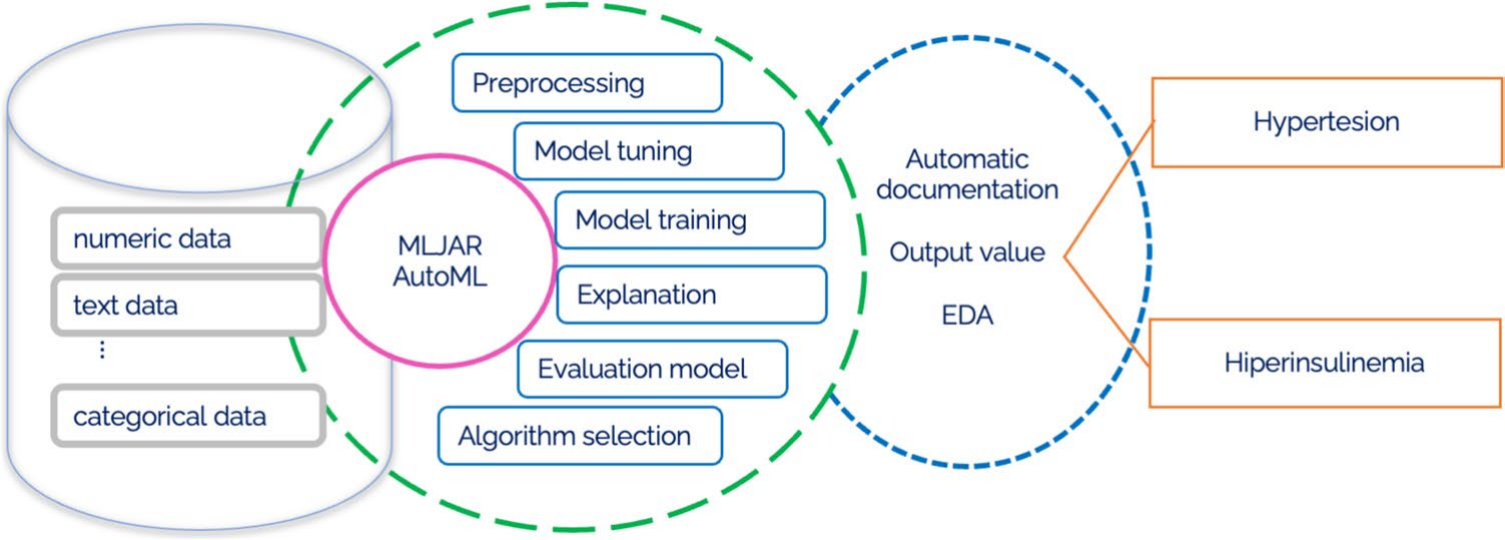
MLJAR pipeline

The third AutoML framework explored in this research is MLJAR, an open-source platform distinguished by its innovative features, notably in the realms of advanced feature creation and selection, which sets it apart from competing AutoML frameworks. These attributes, along with its competitive performance relative to other AutoML platforms, underscore MLJAR’s prominence in the field [55]. Similar to AutoGluon and H2O AutoML, MLJAR does not deploy complex algorithms for solving the Combined Algorithm Selection and Hyperparameter Optimization (CASH) problem. Instead, it adopts a strategy of utilizing a specified array of models along with a predetermined hyperparameter set that is subject to optimization. A random search methodology is employed within this defined space to identify the optimal hyperparameter configurations for the models. MLJAR supports comprehensive automatic data preprocessing, paralleling the functionalities seen in AutoGluon. This includes both model-agnostic and model-specific preprocessing techniques. The framework is capable of automatically handling missing data, as well as categorizing and processing categorical, date/time, and text data, requiring users merely to supply a data frame. MLJAR proceeds with both model-agnostic and model-specific preprocessing autonomously. A distinctive capability not found in AutoGluon or H2O AutoML, yet present in MLJAR, is the feature generation feature, notably through its "Golden Features" functionality. This process entails the synthesis of new features through operations such as addition, subtraction, division, and multiplication among existing features, generating up to 250,000 new features. Subsequently, each feature is evaluated for its informativeness via a dedicated decision tree, culminating in the selection of up to 50 new features. These selected features are then incorporated into the dataset for subsequent model training, further enhancing MLJAR’s analytical capacity [55]. Distinct from AutoGluon and H2O AutoML, MLJAR introduces an innovative approach to automatic feature selection. This method involves the incorporation of a randomly generated numerical feature, uniformly distributed between 0 and 1, into the dataset. Subsequently, the model demonstrating the best performance up to that point is trained on the dataset, now augmented with this random variable. Post-training, the permutation feature importance score is computed to evaluate the informativeness of each variable within the model’s final predictions. Features exhibiting a lower importance score than that of the random feature, indicating inferior informativeness, are eliminated. The dataset, purged of these less informative features, is then used to retrain the top-performing models from each category. Progressing through the MLJAR AutoML pipeline, a hill-climbing algorithm is applied to a selection of the most efficacious models to further refine their performance. This algorithm iteratively adjusts a single hyperparameter at a time, comparing the performance of the modified model against its predecessor, thereby methodically enhancing model efficacy through incremental hyperparameter optimization. The culmination of the MLJAR AutoML process involves the automatic generation of ensemble and stacked ensemble models, aimed at amplifying the collective performance of the models. To construct and ascertain the most optimal stacked ensemble models, MLJAR employs a greedy algorithm, representing the terminal phase of the MLJAR AutoML pipeline [55]. This comprehensive approach underscores MLJAR’s dedication to maximizing model performance through a meticulous and innovative methodological framework. An overview of the MLJAR pipeline can be seen in Fig. 5.

**Fig. 6:**
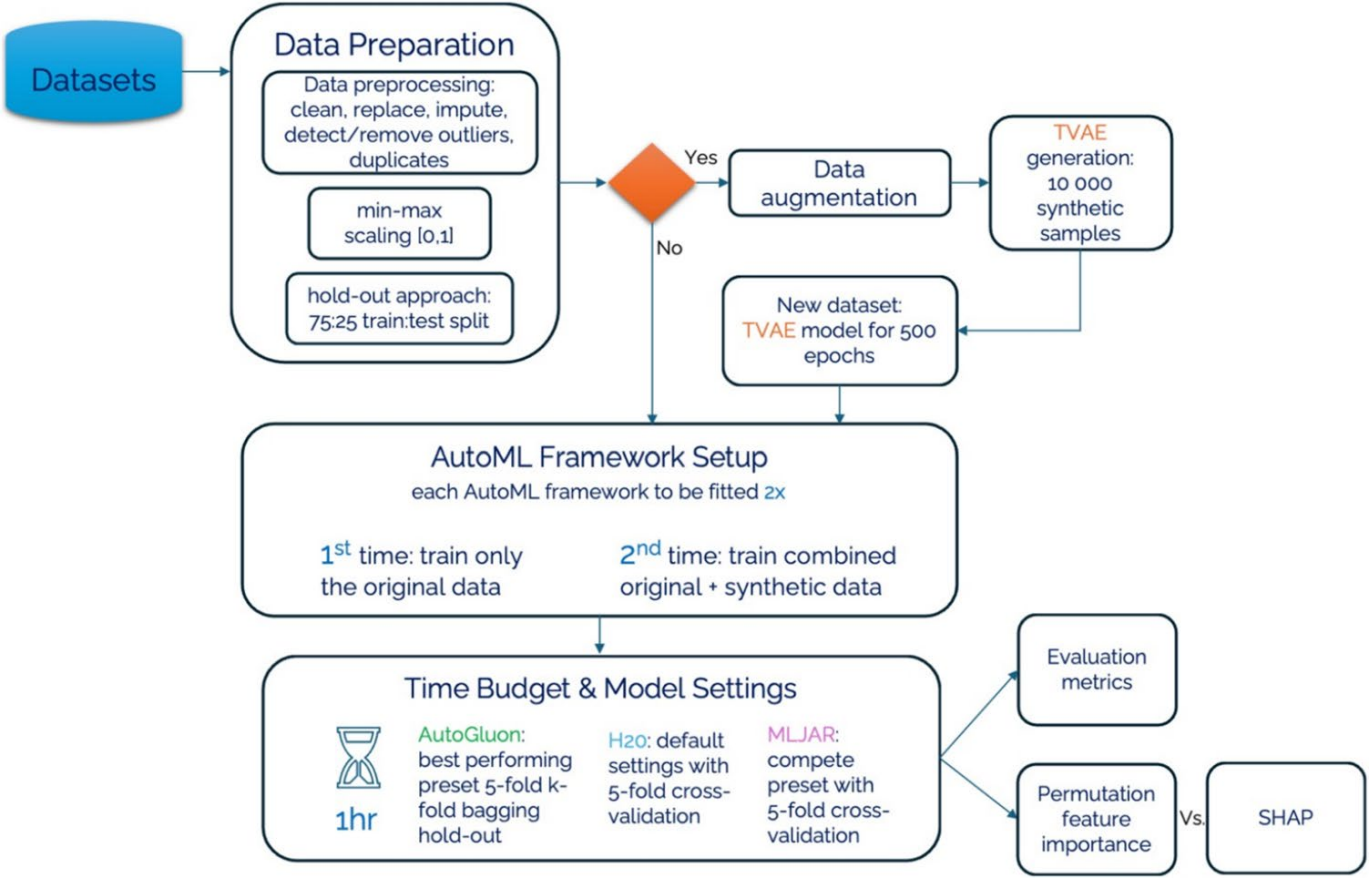
All three AutoML frameworks methodology pipeline

For an enhanced perspective on these AutoML frameworks, we are presenting a methodology pipeline in Fig. 6 and providing a high-level pseudocode for each, to facilitate a straightforward and clear comprehension of these systems.

**Algorithm 1:**
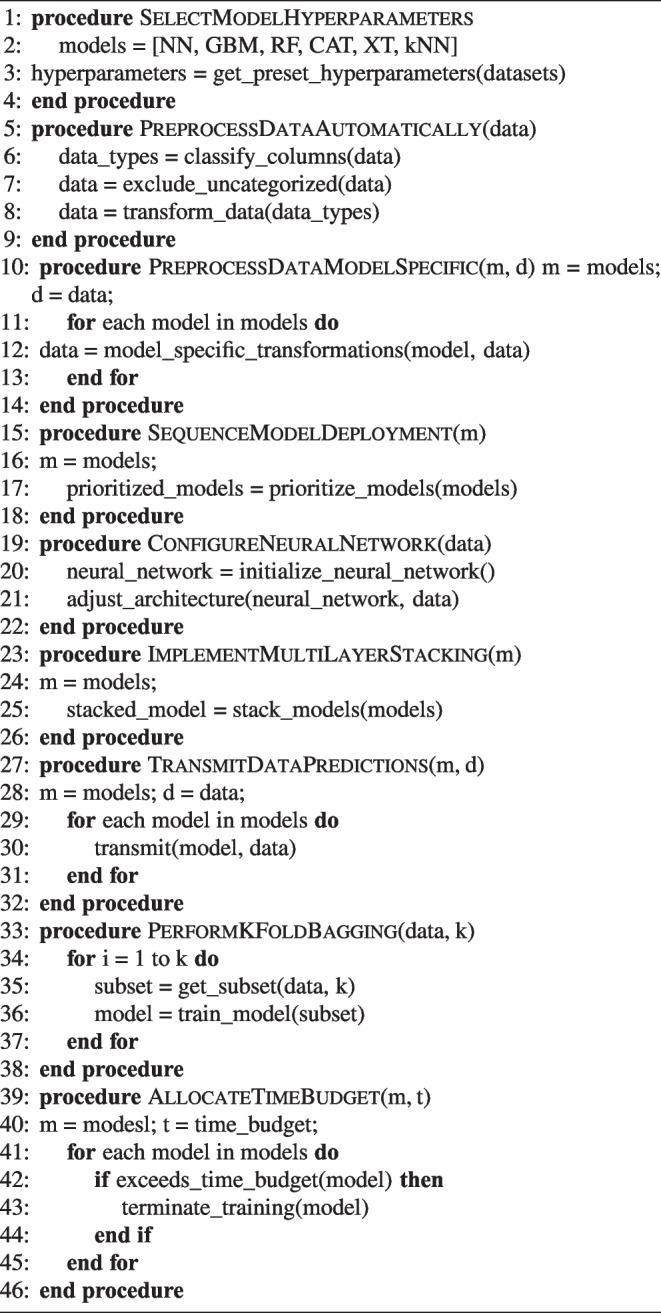
AutoML Process with AutoGluon Framework.

**Algorithm 2:**
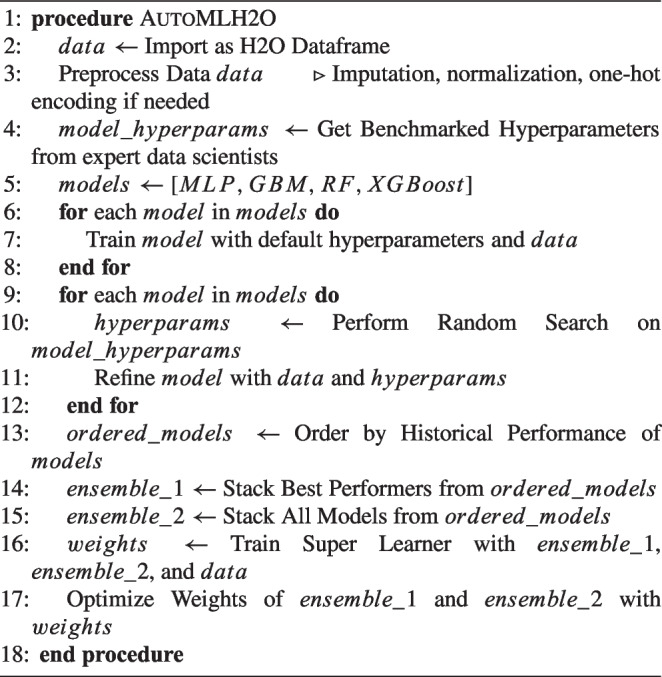
AutoML Process Using H2O Framework.

**Algorithm 3:**
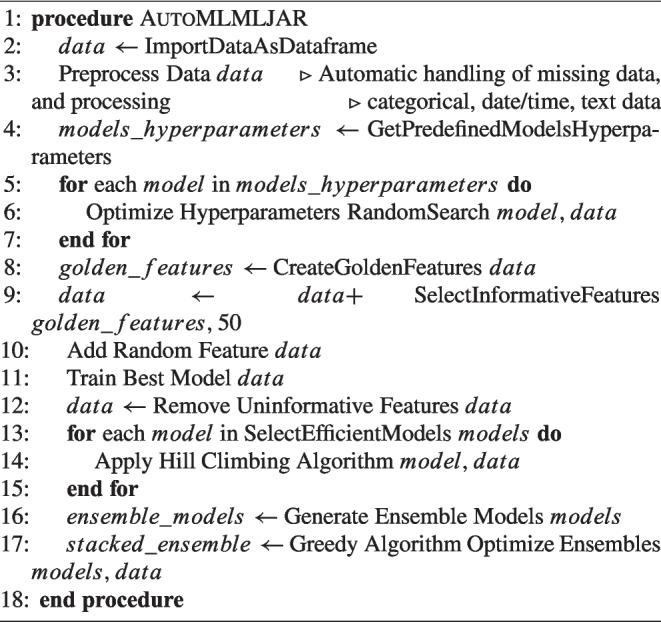
AutoML Process with MLJAR Framework.

## Results

In this section we will elaborated on the results obtained in our experiment. Firstly, it is worth noting that we compared the results of original data and the new dataset constructed using TVAE that generated 10000 to supplement the training data. TVAE model was trained in 500 epochs in total and each AutoML framework was trained twice. Once with only the original training data and the second time using the original training data and the synthetic data combined. Moreover, AutoML pipelines distinguish themselves from traditional data science approaches by training, tuning, and stacking multiple models simultaneously, as opposed to selecting and tuning hyperparameters for individual models. With regard to the Combined Algorithm Selection and Hyperparameter optimization (CASH) problem, AutoML pipelines generally require more training time than conventional methods. These frameworks need a predetermined time limit to terminate the algorithm since training could otherwise continue indefinitely. For this study, a maximum time allowance of one-hour (*1hr window*) has been established for different AutoML frameworks, a duration that benchmarks have indicated to be ample for satisfactory performance of AutoML systems [10, 11, 26, 39]. This one-hour setting is also considered the standard for the AutoML frameworks discussed in this paper. Every AutoML framework has tunable parameters that influence its behavior, such as the selection of models to be trained. AutoGluon, for instance, provides various presets that balance between performance and computational expense. The optimal preset for this study has been selected to provide full functionality and minimize the likelihood of overfitting by enabling 5-fold cross-validation with bagging. The H2O AutoML framework operates solely on default settings, without any parameters that adjust the balance between performance and computational cost. It employs 5-fold cross-validation to mitigate the risk of overfitting. Similarly, MLJAR offers presets that navigate the performance-cost balance. These include ’Explain’, which focuses on speed and model explainability, ’Perform’ for practical applications balancing cost and efficacy, and ’Compete’ for optimal performance, which is the selected preset for this research, with 5-fold cross-validation to fine-tune the models.

### AutoGluon

AutoGluon was able to finish faster than the maximum specified time limit, using the following models for hypertension classification task: NN (Neural Network), GBM (light GBM boosted trees), RF (Random Forest), CAT (Cat Boost boosted trees), KNN (K-Nearest Neighbors), and XT (Extremely Randomized Trees). Within the range 11-13 minutes the AutoGluon pipeline had finished training and produced all the models. During training it used 5 fold bagging, repeated 20 times in order to prevent overfitting. For Scenario 1, weighted ensemble of GBM and XT both achieved the highest scores of 0.909 and 0.916 on train and test data, respectively. They also scored perfectly in precision and nearly perfectly in recall and AUC. NN also performed well with an accuracy of 0.99 and the second-highest F1 score of 0.95. For Scenario 2, NN and GBM algorithms have the highest accuracy of 0.98 and 0.99 scores, respectively. GBM shows a consistent performance with high precision, recall, and AUC metrics. Overall, GBM and XT stand out with high performance across both scenarios, while KNN seems to be the least accurate. The metrics are generally high, suggesting that the models are well-fitted with good generalization capabilities on test scenarios. Precision is consistently high across all algorithms and scenarios, indicating a low false-positive rate. The AUC scores are also high, indicating a good measure of separability achieved by the models. In the results provided, we can see that for each algorithm, the performance in Scenario 1 is generally better than in Scenario 2 when using the AutoGluon tool. For Neural Networks (NN), the scores drop from 0.876 in Scenario 1 to 0.845 in Scenario 2 on train data. The F1 Score, Precision, Recall, and AUC also decrease, though only slightly for the latter three metrics. The GBM shows a slight decrease in train scores from 0.909 to 0.872 and a similar trend is seen in the F1 Score, although the drop in the AUC is marginal. The RF algorithm experiences a decrease in accuracy from 0.94 to 0.93 between Scenarios 1 and 2. The decrease in other metrics like the F1 Score, Precision, Recall, and AUC is also noticeable. For the CAT algorithm, there is also a minor decrease in accuracy from 0.94 to 0.93 and similar declines across the other metrics, which suggest a slight decline in performance. The KNN algorithm shows a reduction in accuracy from 0.90 to 0.88, and the F1 Score, Precision, Recall, and AUC also decrease, indicating a weaker performance in Scenario 2. Lastly, the XT algorithm shows a slight decrease in accuracy from 1.00 to 0.99, with the F1 Score, Precision, Recall, and AUC metrics showing a similar slight decline. Overall, across all algorithms, there is a consistent pattern where Scenario 1 yields better results than Scenario 2. This suggests that the conditions of Scenario 1, whether they are due to the nature of the data or the specific parameters of the test scenario, are more conducive to model performance than those in Scenario 2, Table 3.

**Table 3:** AutoGluon results for hypertension task

Tool	Scenario	Algorithms	Train	Test	Accuracy	F1 Score	Precision	Recall	AUC
AutoGluon	1	NN	0.876	0.802	0.99	0.95	0.99	0.91	0.98
	2	NN	0.845	0.855	0.98	0.93	0.97	0.90	0.96
	1	GBM	0.909	0.916	1.00	0.97	1.00	0.94	0.98
	2	GBM	0.872	0.874	0.99	0.96	0.98	0.93	0.97
	1	RF	0.813	0.827	0.94	0.93	0.96	0.91	0.96
	2	RF	0.782	0.815	0.93	0.92	0.96	0.89	0.95
	1	CAT	0.807	0.810	0.94	0.94	0.97	0.90	0.97
	2	CAT	0.784	0.803	0.93	0.92	0.96	0.89	0.95
	1	KNN	0.762	0.771	0.90	0.88	0.96	0.84	0.94
	2	KNN	0.743	0.753	0.88	0.87	0.95	0.84	0.93
	1	XT	0.909	0.916	1.00	0.97	1.00	0.94	0.98
	2	XT	0.872	0.874	0.99	0.96	0.98	0.93	0.97

Scenario 1: hypertension—original data;

Scenario 2: hypertension—synthetic data

For hyperinsulinemia classification task the same models were used and again within 15 minutes the AutoGluon pipeline had finished training and produced all the models. During training it used 5 fold bagging, repeated 20 times in order to prevent overfitting. For NN, Scenario 2 actually has a higher Accuracy and F1 Score than Scenario 1, unusual when compared to the other algorithms. Specifically, the Accuracy improves from 0.92 to 0.96, and the F1 Score decreases slightly from 0.93 to 0.92. This indicates that for NN, Scenario 2 might have been more aligned with the strengths of this algorithm or the data was more suitable for NN to generalize. However, for the GBM, the performance is better in Scenario 1 with the highest Accuracy of 0.99 and F1 Score of 0.96 noted in the table. In Scenario 2, these scores are slightly lower at 0.97 and 0.94, respectively. RF shows better results in Scenario 1 as well, with an Accuracy of 0.94 and an F1 Score of 0.94, compared to 0.91 and 0.90 in Scenario 2. The CAT algorithm also follows this trend with Scenario 1 outperforming Scenario 2. Scenario 1 has an Accuracy of 0.96 and an F1 Score of 0.93, while Scenario 2 has a lower Accuracy of 0.94 and F1 Score of 0.90. For KNN, Scenario 1 has a marginally better Accuracy of 0.83 and F1 Score of 0.80, whereas Scenario 2 shows a slight decrease with an Accuracy of 0.81 and F1 Score of 0.77. XT reflects a similar pattern where Scenario 1 has an Accuracy of 0.97 and F1 Score of 0.95, which is better than Scenario 2’s Accuracy of 0.96 and F1 Score of 0.94. Overall, with the exception of the NN, Scenario 1 tends to exhibit superior performance over Scenario 2 across most algorithms. This suggests that the conditions or the data in Scenario 1 are more conducive to these algorithms, possibly due to better feature alignment, less noise in the data, or other factors that could affect model performance. The Precision, Recall, and AUC metrics generally follow the same trend as Accuracy and F1 Score for both scenarios across all algorithms, Table 4.

**Table 4:** AutoGluon results for hyperinsulinemia task

Tool	Scenario	Algorithms	Train	Test	Accuracy	F1 Score	Precision	Recall	AUC
AutoGluon	1	NN	0.825	0.794	0.92	0.93	0.97	0.88	0.96
	2	NN	0.833	0.841	0.96	0.92	0.94	0.90	0.95
	1	GBM	0.910	0.917	0.99	0.96	0.99	0.93	0.96
	2	GBM	0.887	0.893	0.97	0.94	0.96	0.91	0.95
	1	RF	0.822	0.803	0.94	0.94	0.92	0.90	0.93
	2	RF	0.780	0.788	0.91	0.90	0.94	0.86	0.91
	1	CAT	0.804	0.811	0.96	0.93	0.97	0.91	0.96
	2	CAT	0.772	0.774	0.94	0.90	0.93	0.87	0.93
	1	KNN	0.754	0.763	0.83	0.80	0.92	0.79	0.89
	2	KNN	0.741	0.744	0.81	0.77	0.80	0.74	0.86
	1	XT	0.834	0.844	0.97	0.95	0.98	0.92	0.97
	2	XT	0.821	0.835	0.96	0.94	0.97	0.91	0.95

1. Scenario 1: hyperinsulinemia—original data;

2. Scenario 2: hyperinsulinemia—synthetic data

### H20

H20 was able to finish faster than the maximum specified time limit, using the following models for hypertension classification task: MLP (Multi Layer Perceptron), XGBoost (Extreme Gradient boosting), GBM (lightGBM boosted trees), RF (Random Forest). AutoH2O has a different performance when compared to AutoGluon. Where AutoGluon trained few models with set hyperparameters, H2O does hyperparamater optimization. Within the given 1 hour time limit, this AutoML framework created and trained a total of 662 models. This is an enormous amount of different models that have been trained and tested with 5-fold cross validation. Due to the fact that there are many models to consider, only the best performing models from each category are selected. Within this framework the top ten models are all stacked models, combining multiple singular models to generate predictions. For Scenario 1, the MLP algorithm achieves the highest Accuracy and F1 Score at 1.00 and 0.98, respectively, with perfect scores in Precision and Recall. In Scenario 2, the performance slightly declines to an Accuracy of 0.98 and an F1 Score of 0.97. The XGBoost in Scenario 1 has an Accuracy of 0.95 and an F1 Score of 0.94, which are notably higher than in Scenario 2, where the Accuracy drops to 0.94 and the F1 Score to 0.92. The GBM algorithm shows an Accuracy of 0.94 and an F1 Score of 0.93 in Scenario 1, both of which are higher than in Scenario 2, where the Accuracy is 0.92 and the F1 Score is 0.90. For the RF algorithm, there is a small difference between the two scenarios. Scenario 1 shows an Accuracy of 0.89 and an F1 Score of 0.87, while Scenario 2 has an Accuracy of 0.84 and an F1 Score of 0.85. Comparing Scenario 1 and Scenario 2, it is evident that all algorithms performed better in Scenario 1. The decline in metrics from Scenario 1 to Scenario 2 suggests that the conditions, such as the dataset or parameter settings in Scenario 2, might be more challenging for the algorithms, impacting their ability to predict as effectively as in Scenario 1. This trend is consistent across all algorithms tested with the H2O framework, with Scenario 1 yielding better results in terms of Accuracy, F1 Score, Precision, Recall, and AUC Table 5.

**Table 5:** H20 results for hypertension task

Tool	Scenario	Algorithms	Train	Test	Accuracy	F1 Score	Precision	Recall	AUC
H20	1	MLP	0.914	0.925	1.00	0.98	1.00	0.95	0.99
	2	MLP	0.892	0.904	0.98	0.97	0.98	0.94	0.97
	1	XG Boost	0.853	0.860	0.95	0.94	0.96	0.92	0.95
	2	XG Boost	0.792	0.807	0.94	0.92	0.96	0.88	0.94
	1	GBM	0.827	0.814	0.94	0.93	0.97	0.91	0.95
	2	GBM	0.776	0.794	0.92	0.90	0.93	0.88	0.93
	1	RF	0.742	0.761	0.89	0.87	0.94	0.83	0.92
	2	RF	0.738	0.742	0.84	0.85	0.93	0.81	0.90

Scenario 1: hypertension—original data;

Scenario 2: hypertension—synthetic data

For hyperinsulinemia task, in Scenario 1, the MLP performs exceptionally well with an Accuracy of 0.99 and an F1 Score of 0.96. Precision, Recall, and AUC are also high, indicating a robust predictive performance. Comparatively, in Scenario 2, the MLP still performs strongly but shows a slight decrease across all metrics, with an Accuracy of 0.96 and an F1 Score of 0.95, which could suggest a more challenging dataset or different parameters that affect the model’s performance. The XG Boost also experiences a decline in performance from Scenario 1 to Scenario 2. In Scenario 1, it achieves an Accuracy of 0.95 and an F1 Score of 0.93. In Scenario 2, while the F1 Score appears to be still high at 0.91, with the Accuracy listed as 0.93, which is a drop from Scenario 1. For GBM, there is a noticeable performance decrease from Scenario 1 to Scenario 2. Scenario 1 shows an Accuracy of 0.92 and an F1 Score of 0.92, whereas Scenario 2 shows reduced Accuracy and F1 Score at 0.85 and 0.84, respectively. RF algorithm’s results mirror this downward trend. In Scenario 1, Accuracy is 0.87 and the F1 Score is 0.89, and both metrics decrease in Scenario 2, with Accuracy of 0.85 and F1 Score to 0.85 Table 6.

**Table 6:** H20 results for hyperinsulinemia task

Tool	Scenario	Algorithms	Train	Test	Accuracy	F1 Score	Precision	Recall	AUC
H20	1	MLP	0.923	0.928	0.99	0.96	0.99	0.93	0.99
	2	MLP	0.897	0.901	0.96	0.95	0.97	0.93	0.96
	1	XG Boost	0.855	0.862	0.95	0.93	0.96	0.91	0.95
	2	XG Boost	0.783	0.977	0.93	0.91	0.95	0.89	0.93
	1	GBM	0.837	0.844	0.92	0.92	0.94	0.91	0.94
	2	GBM	0.789	0.790	0.85	0.84	0.88	0.82	0.85
	1	RF	0.753	0.767	0.87	0.89	0.91	0.87	0.88
	2	RF	0.731	0.740	0.85	0.85	0.87	0.82	0.84

Scenario 1: hyperinsulinemia—original data;

Scenario 2: hyperinsulinemia—synthetic data

### MLJAR

Similarly to AutoGluon and H2O AutoML, the performance of MLJAR on synthetic data was not competitive with the real data. MLJAR was able to obtain the performance in a range with H2O AutoML. Scenario 1 consistently shows better results across all the algorithms when compared to Scenario 2. For example, RF has an Accuracy of 0.91 and an F1 Score of 0.89 in Scenario 1, which decreases to 0.89 and 0.87, respectively, in Scenario 2. This pattern of a slight decrease from Scenario 1 to Scenario 2 is observed in all the algorithms tested. The Light GBM algorithm is the top performer in Scenario 1 with an impressive Accuracy of 0.1.00 and an F1 Score of 0.98. Even though there’s a drop in Scenario 2, it still maintains high performance with an Accuracy of 0.99 and an F1 Score of 0.96. XG Boost also sees a decrease from Scenario 1 to Scenario 2, with the Accuracy dipping from 0.95 to 0.94 and the F1 Score from 0.94 to 0.92. The results of CAT model show a similar decline, with Scenario 1 having an Accuracy of 0.94 and an F1 Score of 0.93, while Scenario 2 shows Accuracy at 0.93 and an F1 Score at 0.92. NN, which has lower overall performance metrics compared to the other algorithms, still experiences a slight reduction in Scenario 2 with an Accuracy and F1 Score going from 0.91 and 0.89 in Scenario 1 to 0.89 and 0.87, respectively Table 7.

**Table 7:** MLJAR results for hypertension task

Tool	Scenario	Algorithms	Train	Test	Accuracy	F1 Score	Precision	Recall	AUC
MLJAR	1	RF	0.772	0.781	0.91	0.89	0.97	0.85	0.94
	2	RF	0.753	0.770	0.89	0.87	0.95	0.84	0.93
	1	Light GBM	0.910	0.914	1.00	0.98	1.00	0.95	0.99
	2	Light GBM	0.893	0.895	0.99	0.96	0.98	0.94	0.97
	1	XG Boost	0.833	0.840	0.95	0.94	0.96	0.92	0.96
	2	XG Boost	0.799	0.801	0.94	0.92	0.96	0.91	0.94
	1	Cat Boost	0.805	0.811	0.94	0.93	0.97	0.90	0.97
	2	Cat Boost	0.772	0.785	0.93	0.92	0.96	0.88	0.95
	1	NN	0.754	0.767	0.91	0.89	0.95	0.85	0.95
	2	NN	0.753	0.764	0.89	0.87	0.93	0.84	0.93

1. Scenario 1: hypertension—original data;

2. Scenario 2: hypertension—synthetic data

For hyperinsulinemia task, the results of RF are better in Scenario 1 with an Accuracy of 0.93 and an F1 Score of 0.90, compared to Scenario 2, where these metrics are 0.89 and 0.87, respectively. This pattern of higher performance in Scenario 1 is consistent across the other algorithms as well. Light GBM stands out with high scores in both scenarios, particularly in Scenario 1 with an Accuracy of 0.99 and an F1 Score of 0.97. Even in Scenario 2, Light GBM performs well, marking an Accuracy of 0.98 and an F1 Score of 0.95, indicating a relatively smaller performance drop compared to other algorithms. XG Boost shows a significant difference between the two scenarios. In Scenario 1, it scores an Accuracy of 0.96 and an F1 Score of 0.95, which are quite robust. However, in Scenario 2, there is a marked decrease with Accuracy falling to 0.93 and F1 Score to 0.91. Cat Boost also exhibits a drop in Scenario 2 but maintains relatively strong performance metrics in both scenarios. It has an Accuracy of 0.94 and an F1 Score of 0.93 in Scenario 1, while Scenario 2 shows a slight decrease to 0.88 Accuracy and 0.84 F1 Score. NN show the least variability between scenarios. In Scenario 1, NN achieves an Accuracy of 0.83 and an F1 Score of 0.83. Scenario 2 has nearly identical results with an Accuracy of 0.82 and an F1 Score of 0.84 Table 8.

**Table 8:** MLJAR results for hyperinsulinemia task

Tool	Scenario	Algorithms	Train	Test	Accuracy	F1 Score	Precision	Recall	AUC
MLJAR	1	RF	0.784	0.788	0.93	0.90	0.94	0.87	0.94
	2	RF	0.762	0.774	0.89	0.87	0.92	0.84	0.89
	1	Light GBM	0.904	0.917	0.99	0.97	0.99	0.96	0.99
	2	Light GBM	0.897	0.902	0.98	0.95	0.98	0.94	0.97
	1	XG Boost	0.845	0.850	0.96	0.95	0.96	0.93	0.96
	2	XG Boost	0.714	0.725	0.93	0.91	0.94	0.87	0.94
	1	Cat Boost	0.815	0.823	0.94	0.93	0.96	0.90	0.95
	2	Cat Boost	0.782	0.790	0.88	0.84	0.97	0.82	0.84
	1	NN	0.734	0.747	0.83	0.83	0.85	0.80	0.83
	2	NN	0.733	0.748	0.82	0.84	0.84	0.81	0.82

Scenario 1: hyperinsulinemia—original data;

Scenario 2: hyperinsulinemia—synthetic data

### AutoML Models interpretability

**Fig. 7:**
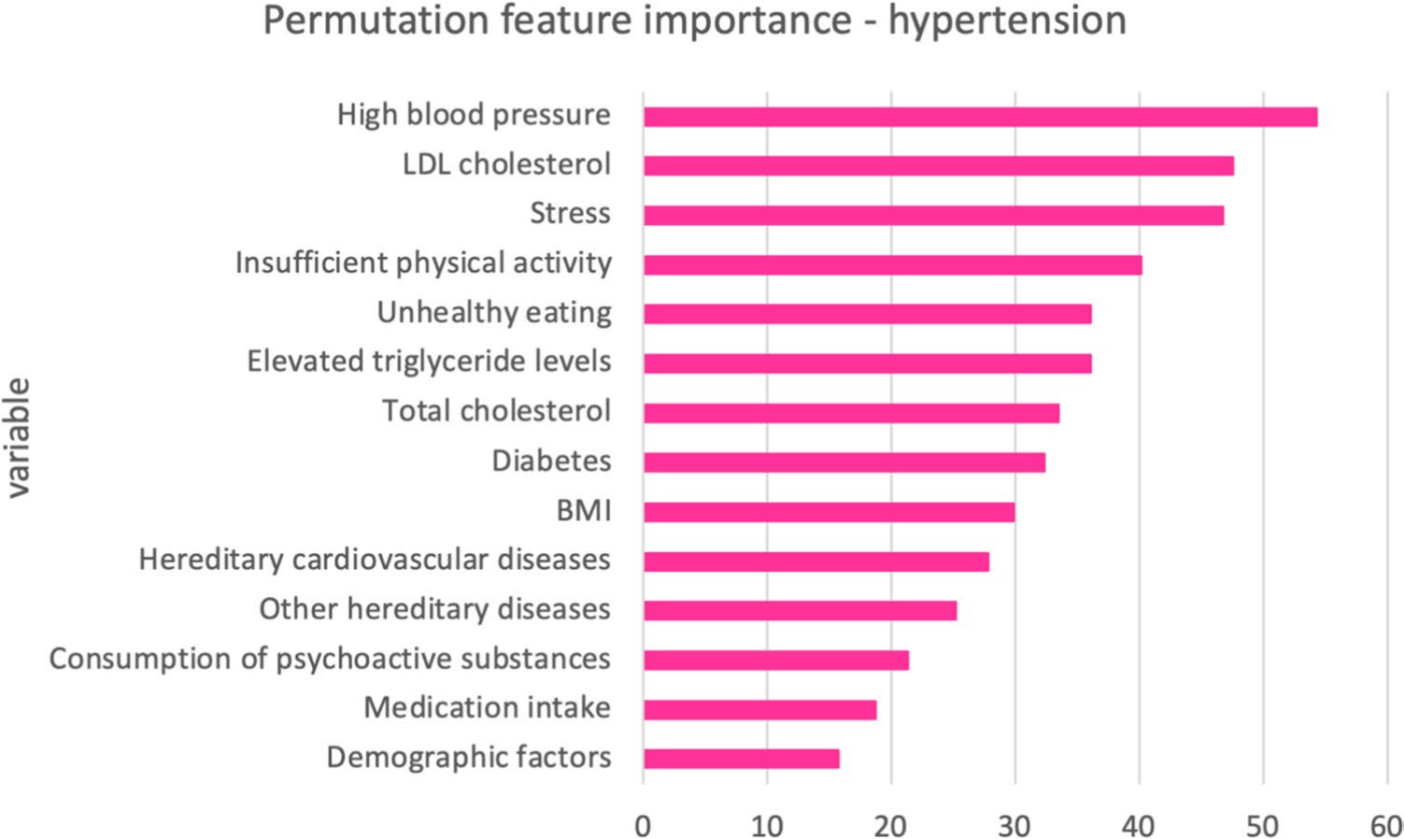
Permutation feature importance for weighted ensemble models in AutoGluon—hypertension task

**Fig. 8:**
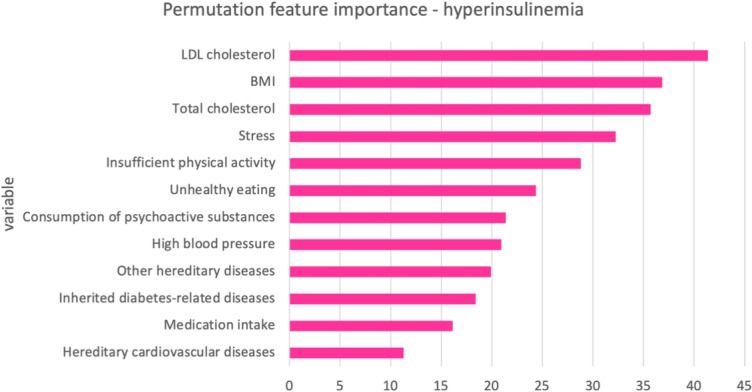
Permutation feature importance for weighted ensemble models in AutoGluon—hyperinsulinemia task

To calculate permutation feature importance for the best-performing models from an AutoML framework, it’s important to note that we will demonstrate the calculation of these scores specifically for the top ensemble models within the AutoGluon framework. Therefore, we will present these values for the AutoGluon framework using real data. This means the reported feature importance scores are only applicable to the real data, not to the synthetic data that has been generated. When examining the feature importance scores for the top 14 variables in hypertension and the top 12 variables in hyperinsulinemia, differences can be observed both in which variables are important and in their respective effects. In a given model, the sum of all feature importance values equals 1, with each score representing the relative importance in comparison to all features. Additionally, in the AutoGluon model, the relative importance of the top features tends to be more uniformly distributed. According to Fig. 7. for hypertension, High blood pressure is shown to be the most significant factor, followed by LDL cholesterol and stress. Other notable variables influencing hypertension include insufficient physical activity, unhealthy eating, and elevated triglyceride levels, although their importance is less pronounced compared to the top three factors. According to Fig. 8. for hyperinsulinemia, LDL cholesterol is identified as the most critical factor, followed closely by BMI and total cholesterol. Other significant variables include stress, insufficient physical activity, and unhealthy eating, indicating that both lifestyle and biological factors play important roles in influencing hyperinsulinemia.

**Fig. 9:**
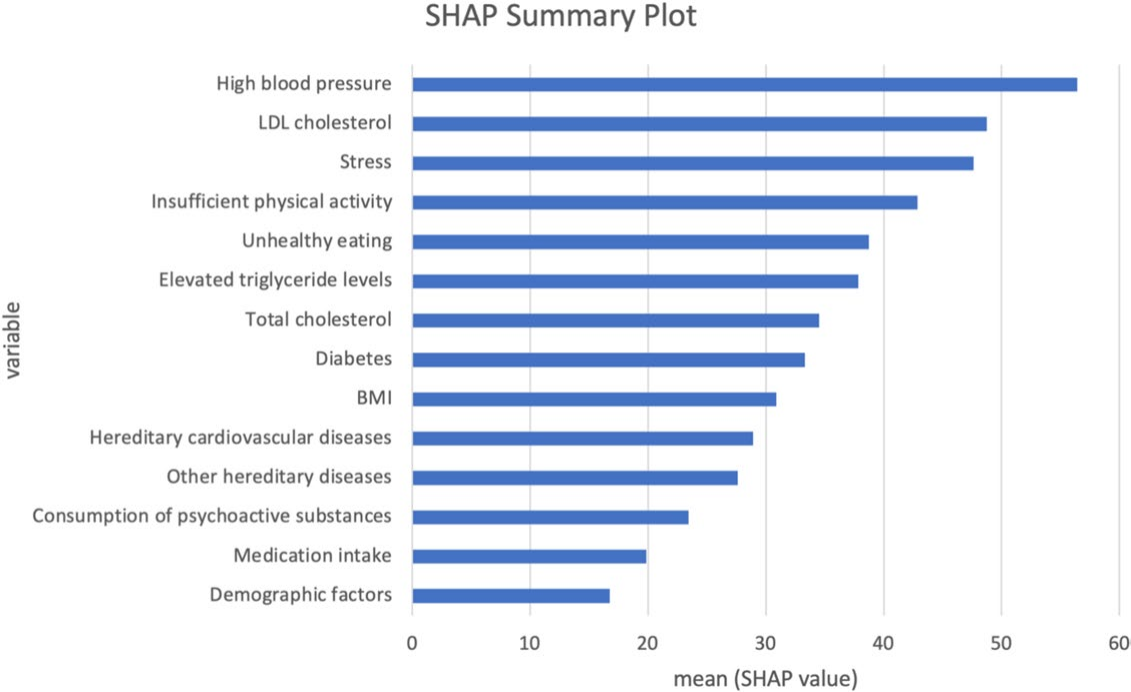
SHAP summary plot for weighted ensemble models in AutoGluon—hypertension task

**Fig. 10:**
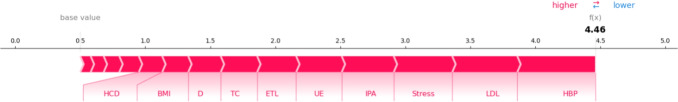
Shap force 1 for hypertension task; where HBP = high blood pressure, IPA = Insufficient physical activity, UE = unhealthy eating, ETL = elevated tryglyceride levels, TC = total cholesterol, D = diabetes, HCD = hereditary cardiovascular disease—hypertension task

**Fig. 11:**
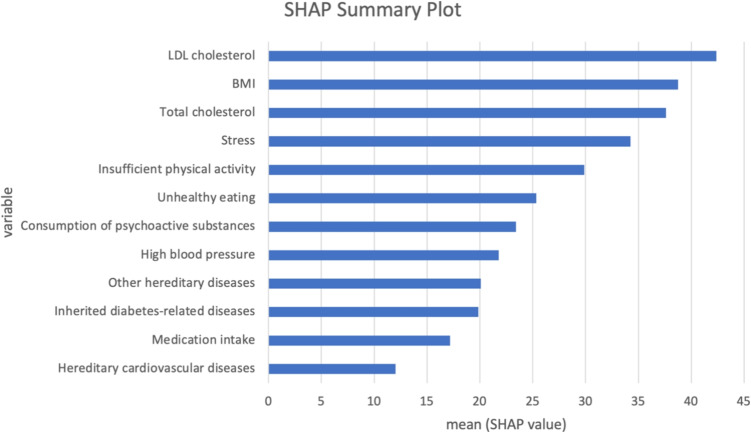
SHAP summary plot for weighted ensemble models in AutoGluon—hyperinsulinemia task

**Fig. 12:**
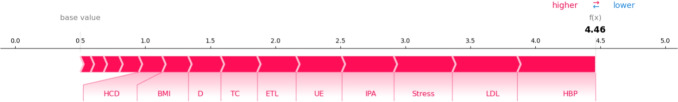
Shap force 2 for hyperinsulinemia task; where HBP = high blood pressure, IPA = Insufficient physical activity, UE = unhealthy eating, ETL = elevated tryglyceride levels, TC = total cholesterol, D = diabetes, HCD = hereditary cardiovascular disease—hyperinsulinemia task

The SHAP summary plot on Fig. 9. for hypertension task presents the average impact of each variable on the model’s output, with the length of the bar representing the mean SHAP value. High blood pressure has the highest influence on the model’s predictions, as indicated by the largest mean SHAP value, followed closely by LDL cholesterol and stress, which exhibit comparable mean SHAP values. While these two factors are slightly lower than high blood pressure, they remain substantial contributors to the model’s output. Variables like insufficient physical activity, unhealthy eating, and elevated triglyceride levels show moderate impact. Lesser but still notable influences come from total cholesterol, diabetes, and BMI. Factors such as hereditary diseases, substance consumption, medication intake, and demographic factors appear to have the least impact according to their mean SHAP values. The SHAP summary plot on Fig. 11. for hyperinsulinemia task shows that LDL cholesterol ranks as the most influential variable, followed by BMI and total cholesterol, suggesting a strong relationship between these factors and the target condition. Variables like stress, insufficient physical activity, and unhealthy eating also contribute significantly, albeit to a lesser extent, while the impact of psychoactive substance consumption, high blood pressure, and other hereditary diseases is moderate. Less influential factors include inherited diabetes-related diseases, medication intake, and hereditary cardiovascular diseases, which have the smallest mean SHAP values. Additionally, from Figs. 10 and 12, it can be seen that for the best-performing AutoML framework, AutoGluon, the contributions of individual features to the predictions for the hypertension and hyperinsulinemia tasks are clearly highlighted. In Fig. 10, which corresponds to the hypertension task, features such as High Blood Pressure (HBP), LDL cholesterol, and Stress exert the most significant influence, pushing the model prediction strongly towards the higher end. Similarly, Fig. 12, representing the hyperinsulinemia task, demonstrates that LDL cholesterol, BMI, and Total Cholesterol (TC) are the dominant contributors, with their SHAP values making the largest positive impact on the model’s output. These force plots visually emphasize the interpretability provided by SHAP, showcasing how the most important risk factors contribute to each task’s predictions and further validating the reliability of the AutoGluon framework in handling healthcare-related datasets. Finally, The inclusion of both global and local SHAP interpretations ensures that our results are aligned with best practices in explainable AI (XAI) within healthcare applications. Since these plots were already part of our previous submission, we emphasize their role in providing transparency to our AutoML framework’s decision-making process. Their presence confirms that the model does not operate as a complete "black box" but instead offers clear, interpretable insights into feature interactions and importance distributions.

### All three frameworks

**Fig. 13:**
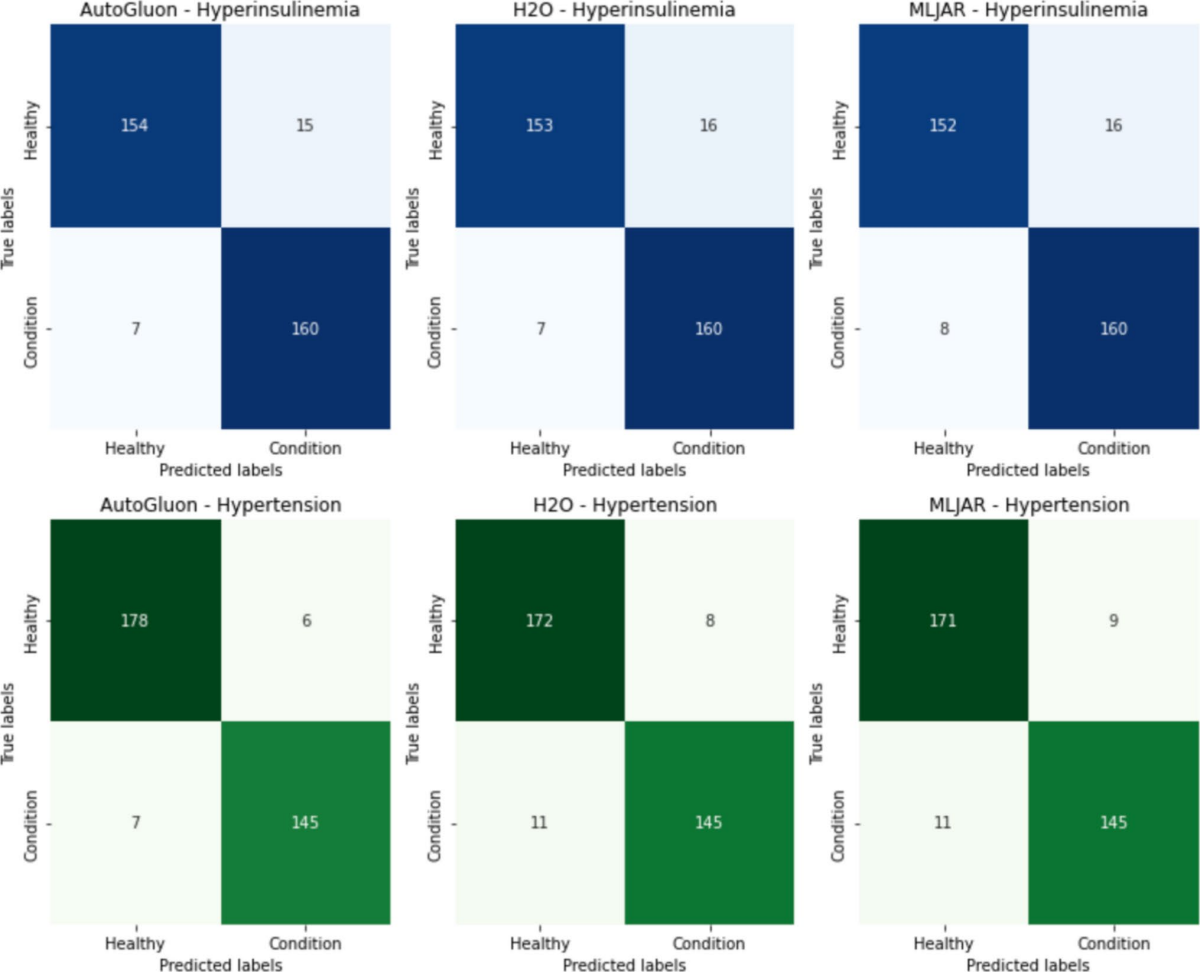
Confusion matrix on real data for Hyperinsulinemia Vs. Hypertension for all three chosen AutoML frameworks

From the Fig. 13 six confusion matrices representing the performance of three different AutoML tools—AutoGluon, H2O, and MLJAR—each applied to classify two different conditions: hyperinsulinemia and hypertension. Confusion matrices are a useful visualization of a model’s performance, displaying the true positives, true negatives, false positives, and false negatives.

For Hyperinsulinemia:

- AutoGluon predicted 154 healthy cases and 160 condition cases correctly, with 15 false negatives and 7 false positives.
- H2O predicted 153 healthy cases and 160 condition cases correctly, with 16 false negatives and 7 false positives.
- MLJAR predicted 152 healthy cases and 160 condition cases correctly, with 16 false negatives and 8 false positives.

For Hypertension:

- AutoGluon predicted 178 healthy cases and 145 condition cases correctly, with 6 false negatives and 7 false positives.
- H2O predicted 172 healthy cases and 145 condition cases correctly, with 8 false negatives and 11 false positives.
- MLJAR predicted 171 healthy cases and 145 condition cases correctly, with 9 false negatives and 11 false positives.

In both conditions, AutoGluon appears to have slightly fewer false negatives and false positives compared to H2O and MLJAR. In the hyperinsulinemia condition, the number of true positives (condition correctly identified) is the same across all tools, while the number of true negatives (healthy correctly identified) is marginally higher for AutoGluon. For the hypertension condition, AutoGluon also has the highest number of true negatives, and all three tools have the same number of true positives.

From Table 9, when observing hypertension classification task, AutoGluon outperforms the other frameworks in terms of mean accuracy, F1 score, precision, recall, and AUC when using the original dataset, with an execution time of just over 11 minutes. The transition to a synthetic dataset increases AutoGluon’s execution time by about 2 minutes while only marginally decreasing its performance metrics. H2O Auto ML’s execution time increases by approximately 2 minutes when working with synthetic data, and this shift also accompanies a more noticeable drop in all performance metrics, especially in mean accuracy and AUC. MLJAR, while taking the longest execution time with both datasets—nearly 18 minutes for the original and over 18 and a half minutes for the synthetic—has a less pronounced performance decrease between the original and synthetic datasets compared to H2O Auto ML. The execution time increases slightly for MLJAR, by less than a minute, when using the synthetic dataset. These observations illustrate that while AutoGluon is the quickest and most accurate, MLJAR is the most stable in terms of performance degradation when shifting from original to synthetic datasets, albeit at the cost of longer execution times Table 9. Additionally, AutoGluon outperforms the other frameworks in terms of mean accuracy, F1 score, precision, recall, and AUC when using the original dataset, with an execution time of just over 11 minutes. The transition to a synthetic dataset increases AutoGluon’s execution time by about 2 minutes while only marginally decreasing its performance metrics. MLJAR, while taking the longest execution time with both datasets—nearly 18 minutes for the original and over 18 and a half minutes for the synthetic—has a less pronounced performance decrease between the original and synthetic datasets compared to H2O Auto ML. The execution time increases slightly for MLJAR, by less than a minute, when using the synthetic dataset. These observations illustrate that while AutoGluon is the quickest and most accurate, MLJAR is the most stable in terms of performance degradation when shifting from original to synthetic datasets, albeit at the cost of longer execution times Table 9.

**Table 9:** All three frameworks compared with mean values of chosen evaluation metrics with execution time for hypertension task

Framework	Ex. Time	mean(Accuracy)	mean(F1)	mean(Precision)	mean(Recall)	mean(AUC)
AutoGluon original	11:03	0.962	0.940	0.980	0.907	0.968
AutoGluon synthetic	13:22	0.950	0.927	0.967	0.897	0.955
H2O Auto ML original	13:57	0.945	0.930	0.9675	0.9025	0.9525
H2O Auto ML synthetic	15:06	0.920	0.910	0.950	0.8775	0.935
MLJAR original	17:59	0.942	0.926	0.970	0.894	0.962
MLJAR synthetic	18:43	0.928	0.908	0.956	0.882	0.944

From Table 10, when observining hyperinsulinemia classification task, AutoGluon with the original dataset is the fastest with an execution time of 9 minutes and 45 seconds, displaying strong performance across all metrics, particularly with a mean accuracy of 0.935. When analyzing synthetic data, AutoGluon’s execution time increases by 1 minute and 38 seconds, and its performance metrics see a slight decrease. H2O Auto ML is a close second in execution time on the original dataset at 12 minutes and 9 seconds and exhibits comparable mean accuracy and a slightly higher mean F1 score than AutoGluon. However, with synthetic data, H2O’s execution time extends by approximately 2 minutes, with a noticeable drop in all performance metrics. MLJAR’s original data execution time stands at 12 minutes and 45 seconds, with mean accuracy and F1 scores just slightly below those of H2O. The switch to synthetic data for MLJAR increases the execution time by over 2 minutes, with a proportionate decrease in performance metrics, showing a resilience similar to AutoGluon but at a slower pace. Overall, while all frameworks show a decline in performance metrics when transitioning from original to synthetic data, AutoGluon is the most time-efficient, especially with the original dataset. The impact on execution time is less for AutoGluon and MLJAR than for H2O Auto ML when handling synthetic data. H2O Auto ML, despite longer execution times, starts with a high baseline in performance metrics on the original dataset but faces a significant drop with synthetic data. MLJAR shows a balanced approach, with moderate execution times and a relatively stable performance decrease when switching datasets. Overally, our AutoGluon framework significantly outperformed literature benchmarks due to multiple methodological enhancements. Firstly, data augmentation using a TVAE expanded the dataset while preserving feature distributions, improving model robustness. Secondly, Principal Component Analysis (PCA) reduced the original 93 features to 12 or 14, isolating the most variance-rich components and mitigating overfitting risks. Thirdly, AutoGluon’s ensemble modeling approach (which integrates deep learning, boosted trees, and stacking techniques) provided additional generalization benefits. These factors collectively contributed to the superior AUC scores of 0.968 for hypertension and 0.945 for hyperinsulinemia, validating the methodological decisions taken in our study.

**Table 10:** All three frameworks compared with mean values of chosen evaluation metrics with execution time for hyperinsulinemia task

Framework	Ex. Time	mean(Accuracy)	mean(F1)	mean(Precision)	mean(Recall)	mean(AUC)
AutoGluon original	09:45	0.935	0.918	0.942	0.888	0.945
AutoGluon synthetic	11:23	0.925	0.895	0.923	0.865	0.925
H2O Auto ML original	12:09	0.933	0.925	0.950	0.905	0.940
H2O Auto ML synthetic	13:47	0.898	0.888	0.918	0.865	0.895
MLJAR original	12:45	0.930	0.916	0.940	0.892	0.934
MLJAR synthetic	14:48	0.900	0.882	0.930	0.856	0.892

## Discussion

In the AutoGluon framework, a variety of models including NN, GBM, RF, CAT, KNN, and XT were utilized for the hypertension classification task, completing training within 11-13 minutes, under the maximum time limit. To mitigate overfitting, the models were trained using 5-fold bagging repeated 20 times. The weighted ensemble of GBM and XT particularly excelled in Scenario 1, showcasing the highest accuracy and F1 scores, alongside near-perfect precision, recall, and AUC metrics. Scenario 2 saw a general dip in performance for all models, yet NN and GBM remained the most accurate. Despite KNN’s lower accuracy, the overall high metrics across models indicate strong fitting and generalization. The consistent higher performance in Scenario 1 implies conditions more favorable to the models than in Scenario 2, suggesting the influence of data nature and test parameters on model efficacy. For the hyperinsulinemia classification task, AutoGluon utilized the same models as in the hypertension task, completing training within a prompt 9 minutes while employing 5-fold bagging repeated 20 times to prevent overfitting. Contrary to other models, the Neural Network (NN) achieved better accuracy and F1 Score in Scenario 2 than in Scenario 1, which is a unique case indicating a possible alignment with NN’s strengths or a dataset particularly well-suited for it. Other models like GBM, RF, CAT, and XT, however, showed a drop in performance when moving to Scenario 2, with GBM maintaining the highest accuracy and F1 score in Scenario 1. This overall trend suggests that the models were more attuned to the conditions of Scenario 1, which may have offered a data environment that was more aligned with their respective strengths, resulting in generally higher precision, recall, and AUC metrics across the board, with the exception of NN.

H2O surpassed the maximum time limit for hypertension classification, generating 662 models within an hour, emphasizing its robust hyperparameter optimization compared to AutoGluon’s predefined settings. For example, in Scenario 1, for hypertension task. the H2O framework’s MLP model topped the performance charts with impressive accuracy and F1 scores of 1.00 and 0.98, respectively, and perfect precision and recall. Although there was a slight decline in Scenario 2 for the MLP, the accuracy and F1 scores remained high at 0.98 and 0.97. XGBoost and GBM also demonstrated higher accuracies in Scenario 1, with a drop in Scenario 2, suggesting it may present a more complex challenge for the models. Consistent with this, all models from H2O displayed superior results in Scenario 1, with Scenario 2 conditions possibly impacting their predictive capabilities. For hyperinsulinemia, MLP again showed high accuracy and F1 scores in Scenario 1, with a minor performance dip in Scenario 2, while XGBoost saw a significant decrease in accuracy, indicating potential errors or data challenges. Overall, H2O’s results suggest Scenario 1 is more favorable for model performance, with robust precision, recall, and AUC across models, whereas Scenario 2 seems to pose greater challenges.

MLJAR, in line with AutoGluon and H2O, showed diminished performance on synthetic data when compared to real data. Within a similar performance range as H2O AutoML, MLJAR’s best results were consistently seen in Scenario 1 across all algorithms. For instance, in Scenario 1 for hypertension task, even RF demonstrated an Accuracy of 0.91 and an F1 Score of 0.89 in Scenario 1, with a slight drop to 0.89 and 0.87 in Scenario 2. Light GBM was the standout in Scenario 1 with an impressive Accuracy of 1.00 and an F1 Score of 0.98, maintaining high performance even in Scenario 2. XG Boost, CAT, and NN all followed the trend of better performance in Scenario 1, with NN showing the least drop between scenarios. For the hyperinsulinemia task, MLJAR’s algorithms also performed best in Scenario 1, with Light GBM again excelling and NN displaying the smallest difference in performance between scenarios, underscoring the consistent trend across different tasks and data types.

When it comes to computational sustainability, execution times are a crucial aspect of evaluating machine learning frameworks, as shorter times often equate to reduced computational resources and energy consumption. AutoGluon emerges as the leader in efficiency, completing tasks swiftly with the least performance impact when transitioning from real to synthetic datasets. Despite a marginal increase in time, AutoGluon demonstrates robustness and speed, underscoring its viability for scenarios demanding quick turnarounds. H2O Auto ML, while competitive in initial speed and accuracy on real data, experiences a significant extension in execution time with synthetic data, raising concerns about its sustainability in continuous or resource-constrained environments. MLJAR strikes a balance between resilience to data changes and execution time, though its longer processing period suggests a higher resource usage, which may not be ideal for situations where energy conservation is vital.

Permutation feature importance and SHAP values provide insights into the predictive power and influence of individual features within machine learning models. In AutoGluon’s ensemble models, these methods highlight which factors most significantly affect hypertension and hyperinsulinemia predictions using real data. For hypertension, high blood pressure, LDL cholesterol, and stress emerge as the top influencing variables, with physical activity, diet, and triglycerides also playing notable roles. In the context of hyperinsulinemia, LDL cholesterol, BMI, and total cholesterol are identified as primary factors, along with stress and lifestyle habits. The SHAP plots further quantify these impacts, offering a visual representation of each variable’s mean contribution to model output, emphasizing the differential influence across the range of features and providing a clear hierarchy of feature importance for both health conditions.

## Conclusion

In this research, our goal to explore AutoML approach has been taken in order to classify hypertension and hyperinsulinemia conditions among adolescents. In addition the option of synthetic data generation in order to artificially increase the training data has been explored and implemented through the usage of a TVAE. Three AutoML framework, namely AutoGluon, H20, and MLJAR were chosen and have been trained on the data, once with the real training data and once with the synthetic data, with a one hour time budget. The usage of synthetic or real training data had a large impact on model performance. Whilst the expectation was that a larger training set would allow the models to more effectively learn patterns from the data and therefore improve the accuracy, the result was the opposite. The performance of the real training data using AutoML has proven to be promissing. Even when comparing to the state of the art performance that was recently published by [58, 59], there is still a possibility for an improvement. AutoGluon has been able to outperform the state of the art performance slightly. This shows that accessible AutoML frameworks have the potential to provide users with easy, yet powerful, results even when compared to manual training and designing of models. It is worth noting that the incorporation of synthetic data generated via TVAE directly addressed the inherent challenges of the original dataset, including extreme class imbalances, feature sparsity, and low cardinality, which are typical in observational cross-sectional medical datasets. The use of TVAE allowed for the generation of statistically consistent yet novel data points by learning the latent joint probability distributions of the original features. This augmentation improved class representation, reducing overfitting on majority classes and ensuring a more uniform decision boundary. Furthermore, when combined with PCA for dimensionality reduction, the augmented dataset reinforced the retention of critical latent structures in high-dimensional feature spaces, enabling more effective model training without compromising computational efficiency. Finally, it is worth nothing that the 12-minute budget-time constraint was determined through extensive trial-and-error experiments in our lab environment, optimizing the trade-off between computational efficiency and predictive performance. Given the heterogeneity of AutoGluon’s model selection and ensembling processes, training times varied significantly across configurations. Empirical evaluations revealed that beyond 12 minutes, performance improvements plateaued while computational costs increased disproportionately. This constraint aligns with real-world healthcare deployment scenarios, where rapid inference is critical for decision support. By setting this time budget, we demonstrated that state-of-the-art predictive accuracy can be achieved under practical compute constraints, reinforcing the feasibility of AutoML-driven diagnostics in clinical applications.

Training models with both original and synthetic data enhanced their robustness, particularly for underrepresented classes and feature interactions. Synthetic data also provided a controlled framework for evaluating feature importance through SHAP and Permutation Importance, leading to a more granular understanding of the most influential predictors of hypertension and hyperinsulinemia. Despite a slight decline in performance when synthetic data was used in isolation, its inclusion bolstered interpretability, reduced biases, and supported the creation of a more transparent and generalizable diagnostic framework. These benefits align with the research objectives of developing reliable AutoML pipelines tailored to the complexities of real-world healthcare applications. To conclude, AutoGluon, H2O, and MLJAR can be used not only to identify hypertension and hyperinsulinemia but also to identify various non-communicable diseases. These AutoML frameworks are highly flexible and can be adapted to different types of data and problems, including those related to non-communicable diseases such as diabetes, heart disease, cancer, and many others. By using advanced machine learning algorithms, AutoML tools can analyze large and complex medical datasets to identify patterns and associations relevant for diagnosing and predicting various non- communicable diseases. The automation of feature selection, data engineering, model selection, hyperparameter optimization, and model validation processes allows for faster and more efficient achievement of high-performing models, which is crucial for accurate disease identification. Moreover, the interpretability tools provided by these AutoML frameworks help understand the contribution of individual features in predictions, which is important for making informed medical decisions. All these characteristics make AutoML frameworks useful tools for identifying and managing various non-communicable diseases.

### Limitations

The utility of AutoML models presents both promise and challenge. Notably, one of the primary limitations is the interpretability issue stemming from model stacking; complex ensemble methods can obscure the decision-making process, making it difficult to discern how predictions are generated. Despite multiple training iterations, only the most successful models afforded sufficient clarity for detailed feature analysis. When situating this study within the broader landscape of research, it’s critical to acknowledge the constrained scope of data employed. The generalizability of this study—and possibly AutoML at large—is inherently tied to the available hardware and time constraints. With more computational time, better hardware, or a larger dataset, the outcomes could markedly differ. Moreover, the small dataset size in this research might have contributed to overfitting issues, hinting that an expansion of data might not only mitigate this concern but possibly elevate model performance further.

### Future work

Future investigations would benefit from incorporating a more expansive dataset, such as international metadata from electronic health records, rather than relying solely on national data. Such an approach may offer deeper insights and enhance the robustness of the findings. The rapid training capability of AutoML, as demonstrated by the models’ completion within an hour, particularly for small datasets, highlights the frameworks’ efficiency and reduced computational demands. AutoGluon’s standout performance, with completion times of 11 and 9 minutes for the hypertension and hyperinsulinemia tasks respectively, raises questions about its scalability to larger datasets. Interestingly, AutoGluon emerged as both the fastest and the most accurate framework, aligning with benchmarks from recent literature, despite eschewing hyperparameter tuning. This raises speculation about the potential gains other frameworks could achieve with additional resources and time, especially MLJAR, which did not engage in feature selection due to time constraints. The impact of data augmentation on model performance remains a complex area of investigation, as reflected in our results. While synthetic data augmentation was implemented to balance class distributions and increase sample diversity, models trained on augmented data performed slightly worse than those trained on real data. This outcome is consistent with existing literature, where synthetic data can introduce distributional shifts and latent feature noise, potentially weakening the learned representations. We hypothesize that despite TVAE’s ability to generate structurally sound samples, some high-dimensional dependencies within the real dataset were not fully replicated, leading to information loss. Future work will also focus on refining augmentation techniques to optimize the trade-off between increased sample.

## Data Availability

The raw dataset used for this study is under a Non-Disclosure Agreement (NDA) and is therefore not available to the public.

## Code Availability

The code used in this research will be made available upon reasonable request via email of corresponding author.
